# Development and internal-external validation of statistical and machine learning models for breast cancer prognostication: cohort study

**DOI:** 10.1136/bmj-2022-073800

**Published:** 2023-05-10

**Authors:** Ash Kieran Clift, David Dodwell, Simon Lord, Stavros Petrou, Michael Brady, Gary S Collins, Julia Hippisley-Cox

**Affiliations:** 1 Cancer Research UK Oxford Centre, Oxford, UK; 2 Nuffield Department of Primary Care Health Sciences, Radcliffe Primary Care Building, Radcliffe Observatory Quarter, University of Oxford, Oxford OX2 6GG, UK; 3 Nuffield Department of Population Health, University of Oxford, Oxford, UK; 4 Department of Oncology, University of Oxford, Oxford, UK; 5 Centre for Statistics in Medicine, Nuffield Department of Orthopaedics, Rheumatology and Musculoskeletal Sciences, University of Oxford, Oxford, UK

## Abstract

**Objective:**

To develop a clinically useful model that estimates the 10 year risk of breast cancer related mortality in women (self-reported female sex) with breast cancer of any stage, comparing results from regression and machine learning approaches.

**Design:**

Population based cohort study.

**Setting:**

QResearch primary care database in England, with individual level linkage to the national cancer registry, Hospital Episodes Statistics, and national mortality registers.

**Participants:**

141 765 women aged 20 years and older with a diagnosis of invasive breast cancer between 1 January 2000 and 31 December 2020.

**Main outcome measures:**

Four model building strategies comprising two regression (Cox proportional hazards and competing risks regression) and two machine learning (XGBoost and an artificial neural network) approaches. Internal-external cross validation was used for model evaluation. Random effects meta-analysis that pooled estimates of discrimination and calibration metrics, calibration plots, and decision curve analysis were used to assess model performance, transportability, and clinical utility.

**Results:**

During a median 4.16 years (interquartile range 1.76-8.26) of follow-up, 21 688 breast cancer related deaths and 11 454 deaths from other causes occurred. Restricting to 10 years maximum follow-up from breast cancer diagnosis, 20 367 breast cancer related deaths occurred during a total of 688 564.81 person years. The crude breast cancer mortality rate was 295.79 per 10 000 person years (95% confidence interval 291.75 to 299.88). Predictors varied for each regression model, but both Cox and competing risks models included age at diagnosis, body mass index, smoking status, route to diagnosis, hormone receptor status, cancer stage, and grade of breast cancer. The Cox model’s random effects meta-analysis pooled estimate for Harrell’s C index was the highest of any model at 0.858 (95% confidence interval 0.853 to 0.864, and 95% prediction interval 0.843 to 0.873). It appeared acceptably calibrated on calibration plots. The competing risks regression model had good discrimination: pooled Harrell’s C index 0.849 (0.839 to 0.859, and 0.821 to 0.876, and evidence of systematic miscalibration on summary metrics was lacking. The machine learning models had acceptable discrimination overall (Harrell’s C index: XGBoost 0.821 (0.813 to 0.828, and 0.805 to 0.837); neural network 0.847 (0.835 to 0.858, and 0.816 to 0.878)), but had more complex patterns of miscalibration and more variable regional and stage specific performance. Decision curve analysis suggested that the Cox and competing risks regression models tested may have higher clinical utility than the two machine learning approaches.

**Conclusion:**

In women with breast cancer of any stage, using the predictors available in this dataset, regression based methods had better and more consistent performance compared with machine learning approaches and may be worthy of further evaluation for potential clinical use, such as for stratified follow-up.

## Introduction

Clinical prediction models already support medical decision making in breast cancer by providing individualised estimations of risk. Tools such as PREDICT Breast^1^ or the Nottingham Prognostic Index^2^ ^3^ are used in patients with early stage, surgically treated breast cancer for prognostication and selection of post-surgical treatment. Such tools are, however, inherently limited to treatment specific subgroups of patients. Accurate estimation of mortality risk after diagnosis across all patients with breast cancer of any stage may be clinically useful for stratifying follow-up, counselling patients about their expected prognosis, or identifying high risk individuals suitable for clinical trials.^4^

The scope for machine learning approaches in clinical prediction modelling has attracted considerable interest.^5^ ^6^ ^7^ ^8^ ^9^ Some have posited that these flexible approaches might be more suitable for capturing non-linear associations, or for handling higher order interactions without explicit programming.^10^ Others have raised concerns about model transparency,^11^ ^12^ interpretability,^13^ risk of algorithmic bias exacerbating extant health inequalities,^14^ quality of evaluation and reporting,^15^ ability to handle rare events^16^ or censoring,^17^ and appropriateness of comparisons^11^ to regression based methods.^18^ Indeed, systematic reviews have shown no inherent benefit of machine learning approaches over appropriate statistical models in low dimensional clinical settings.^18^ As no a priori method exists to predict which modelling approach may yield the most useful clinical prediction model for a given scenario, frameworks that appropriately compare different models can be used.

Owing to the risks of harm from suboptimal medical decision making, clinical prediction models should be comprehensively evaluated for performance and utility,^19^ and, if widespread clinical use is intended, heterogeneity in model performance across relevant patient groups should be explored.^20^ Given developments in treatment for breast cancer over time, with associated temporal falls in mortality, another key consideration is the transportability of risk models—not just across regions and subpopulations but also across time periods.^21^ Although such dataset shift^22^ is a common issue with any algorithm sought to be deployed prospectively, this is not routinely explored. Robust evaluation is necessary but is non-uniform in the modelling of breast cancer prognostication.^23^ A systematic review identified 58 papers that assessed prognostic models for breast cancer,^24^ but only one study assessed clinical effectiveness by means of a simplistic approach measuring the accuracy of classifying patients into high or low risk groups. A more recent systematic review^25^ appraised 922 breast cancer prediction models using PROBAST (prediction model risk of bias assessment tool)^26^ and found that most of the clinical prediction models are poorly reported, show methodological flaws, or are at high risk of bias. Of the 27 models deemed to be at low risk of bias, only one was intended to estimate the risks of breast cancer related mortality in women with disease of any stage.^27^ However, this small study of 287 women using data from a single health department in Spain had methodological limitations, including possibly insufficient data to fit a model (see supplementary table 1) and uncertain transportability to other settings. Therefore, no reliable prediction model exists to provide accurate risk assessment of mortality in women with breast cancer of any stage. Although we refer to women throughout, this is based on self-reported female sex, which may include some individuals who do not identify as female. 

We aimed to develop a clinically useful prediction model to reliably estimate the risks of breast cancer specific mortality in any woman with a diagnosis of breast cancer, in line with modern best practice. Utilising data from 141 675 women with invasive breast cancer diagnosed between 2000 and 2020 in England from a population representative, national linked electronic healthcare record database, this study comparatively developed and evaluated clinical prediction models using a combination of analysis methods within an internal-external validation strategy.^28^ ^29^ We sought to identify and compare the best performing methods for model discrimination, calibration, and clinical utility across all stages of breast cancer.

## Methods

We evaluated four model building approaches: two regression methods (Cox proportional hazards and competing risks regression) and two machine learning methods (XGBoost and neural networks). The prediction horizon was 10 year risk of breast cancer related death from date of diagnosis. The study was conducted in accordance with our protocol^30^ and is reported consistent with the TRIPOD (transparent reporting of a multivariable prediction model for individual prognosis or diagnosis) guidelines.^31^

### Sample size calculations

Assuming 100 candidate predictor parameters, an annual mortality rate of 0.024 after diagnosis,^32^ and a conservative 15% of the maximal Cox-Snell R^2^, we estimated that the minimum sample size for fitting the regression models was 10 080, with 1452 events, and 14.52 events for each predictor parameter.^33^ ^34^ No standard method exists to estimate minimum sample size for our machine learning models of interest—some evidence, albeit on binary outcome data, suggests that some machine learning methods may require much more data.^35^

### Study population and data sources

The QResearch database was used to identify an open cohort of women aged 20 years and older (no upper age limit) at time of diagnosis of any invasive breast cancer between 1 January 2000 and 31 December 2020 in England. QResearch has collected data from more than 1500 general practices in the United Kingdom since 1989 and comprises individual level linkage across general practice data, NHS Digital’s Hospital Episode Statistics, the national cancer registry, and the Office for National Statistics death registry.

### Patient and outcome definitions

The outcome for this study was breast cancer related mortality within 10 years from the date of a diagnosis of invasive breast cancer. We defined the diagnosis of invasive breast cancer as the presence of breast cancer related Read/Systemised Nomenclature of Medicine – Clinical Terms (SNOMED) codes in general practice records, breast cancer related ICD-10 (international classification of diseases, 10th revision) codes in Hospital Episode Statistics data, or as a patient with breast cancer in the cancer registry (stage >0; whichever occurred first). The outcome, breast cancer death, was defined as the presence of relevant ICD-10 codes as any cause of death (primary or contributory) on death certificates from the ONS register. We excluded women with recorded carcinoma in situ only diagnoses as these are non-obligate precursor lesions and present distinct clinical considerations.^36^ Clinical codes used to define predictors and outcomes are available in the QResearch code group library (https://www.qresearch.org/data/qcode-group-library/ ). Follow-up time was calculated from the first recorded date of breast cancer diagnosis (earliest recorded on any of the linked datasets) to the earliest of breast cancer related death, other cause of death, or censoring (reached end of study period, left the registered general practice, or the practice stopped contributing to QResearch). The status at last follow-up depended on the modelling framework (ie, Cox proportional hazards or competing risks framework). The maximum follow-up was truncated to 10 years, in line with the model prediction horizon. Supplementary table 2 shows ascertainment of breast cancer diagnoses across the linked datasets.

### Candidate predictor parameters

Individual participant data were extracted on the candidate predictor parameters listed in Box 1, as well as geographical region, auxiliary variables (breast cancer treatments), and dates of events of interest. Candidate predictors were based on evidence from the clinical, epidemiological, or prediction model literature.^1^ ^23^ ^37^ ^38^ ^39^ ^40^ The most recently recorded values before or at the time of breast cancer diagnosis were used with no time restriction. Data were available from the cancer registry about cancer treatment within one year of diagnosis (eg, chemotherapy) but without any corresponding date. The intended model implementation (prediction time) would be at the breast cancer multidisciplinary team meeting or similar clinical setting, following initial diagnostic investigations and staging. To avoid information leakage, and since we did not seek model treatment selection within a causal framework,^41^ breast cancer treatment variables were not included as predictors.

Box 1 **Candidate predictor parameters for models**

**Candidate predictor parameters, definitions, and functional forms explored**

Age at breast cancer diagnosis—continuous or fractional polynomial
Townsend deprivation score at cohort entry—continuous or fractional polynomial
Body mass index (most recently recorded before breast cancer diagnosis)—continuous or fractional polynomial
Self-reported ethnicity
Tumour characteristics:
  Cancer stage at diagnosis (ordinal: I, II, III, IV)
  Differentiation (categorical: well differentiated, moderately differentiated, poorly or undifferentiated)
  Oestrogen receptor status (binary: positive or negative)
  Progesterone receptor status (binary: positive or negative)
  Human epidermal growth factor receptor 2 (HER2) status (binary: positive or negative)
  Route to diagnosis (categorical: emergency presentation, inpatient elective, other, screen detected, two week wait)
Comorbidities or medical history on general practice or Hospital Episodes Statistics data (recorded before or at entry to cohort; categorical unless stated otherwise):
  Hypertension
  Ischaemic heart disease
  Type 1 diabetes mellitus
  Type 2 diabetes mellitus
  Chronic liver disease or cirrhosis
  Systemic lupus erythematosus
  Chronic kidney disease (ordinal: none or stage 2, stage 3, stage 4, stage 5)
  Vasculitis
Family history of breast cancer (categorical: recorded in general practice or Hospital Episodes Statistics data, before or at entry to cohort)
Drug use (before breast cancer diagnosis):
  Hormone replacement therapy
  Antipsychotic
  Tricyclic antidepressant
  Selective serotonin reuptake inhibitor
  Monoamine oxidase inhibitor
  Oral contraceptive pill
  Angiotensin converting enzyme inhibitor
  β blocker
  Renin-angiotensin aldosterone antagonists
Age (fractional polynomial terms)×family history of breast cancer
Ethnicity×age (fractional polynomial terms)

Interactions and fractional polynomials were not included in the machine learning models.

Fractional polynomial^42^ terms for the continuous variables age at diagnosis, Townsend deprivation score, and body mass index (BMI) at diagnosis were identified in the complete data. This was done separately for the Cox and competing risks regression models, with a maximum of two powers permitted.

### Missing data

Multiple imputation with chained equations was used to impute missing data for BMI, ethnicity, Townsend deprivation score, smoking status, cancer stage at diagnosis, cancer grade at diagnosis, HER2 status, oestrogen receptor status, and progesterone receptor status under the missing at random assumption.^43^ ^44^ The imputation model contained all other candidate predictors, the endpoint indicator, breast cancer treatment variables, the Nelson-Aalen cumulative hazard estimate,^45^ and the period of cohort entry (period 1=1 January 2000-31 December 2009; period 2=1 January 2010-31 December 2020). The natural logarithm of BMI was used in imputation for normality, with imputed values exponentiated back to the regular scale for modelling. We generated 50 imputations and used these in all model fitting and evaluation steps. Although missing data were observed in the linked datasets used for model development, in the intended use setting (ie, risk estimation at breast cancer multidisciplinary team after a medical history has been taken), the predictors would be expected to be available for all patients.

### Modelling strategy

**Fig 1:**
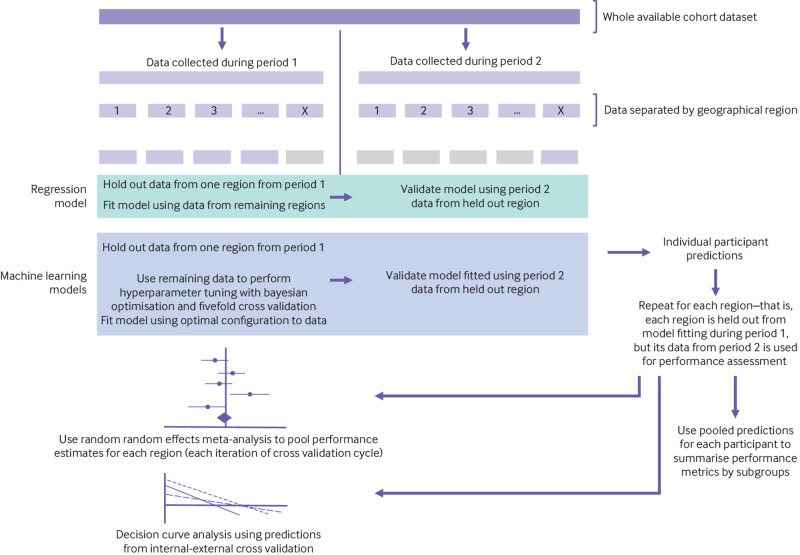
Summary of internal-external cross validation framework used to evaluate model performance for several metrics, and transportability

Models were fit to the entire cohort and then evaluated using internal-external cross validation,^28^ which involved splitting the dataset by geographical region (n=10) and time period (see figure 1 for summary). For the internal-external cross validation, we recalculated follow-up so that those women who entered the study during the first study decade and survived into the second study period had their follow-up truncated (and status assigned accordingly) at 31 December 2009. This was to emulate two wholly temporally distinct datasets, both with maximum follow-up of 10 years, for the purposes of estimating temporal transportability of the models.

### Cox proportional hazards modelling

For the approach using Cox proportional hazards modelling, we treated other (non-breast cancer) deaths as censored. A full Cox model was fitted using all candidate predictor parameters. Model fitting was performed in each imputed dataset and the results combined using Rubin’s rules, and then this pooled model was used as the basis for predictor selection. We selected binary or multilevel categorical predictors associated with exponentiated coefficients >1.1 or <0.9 (at P<0.01) for inclusion, and interactions and continuous variables were selected if associated with P<0.01. Then these were used to refit the final Cox model. The predictor selection approach benefits from starting with a full, plausible, maximally complex model,^46^ and then considers both the clinical and the statistical magnitude of predictors to select a parsimonious model while making use of multiply imputed data.^47^ ^48^ This approach has been used in previous clinical prediction modelling studies using QResearch.^49^ ^50^ ^51^ Clustered standard errors were used to account for clustering of participants within individual general practices in the database.

### Competing risks models

Deaths from other, non-breast cancer related causes represent a competing risk and in this framework were handled accordingly.^30^ We repeated the fractional polynomial term selection and predictor selection processes for the competing risks models owing to potential differential associations between predictors and risk or functional forms thereof. A full model was fit with all candidate predictors, with the same magnitude and significance rule used to select the final predictors.

The competing risks model was developed using jack-knife pseudovalues for the Aalen-Johansen cumulative incidence function at 10 years as the outcome variable^52^—the pseudovalues were calculated for the overall cohort (for fitting the model) and then separately in the data from period 1 and from period 2 for the purposes of internal-external cross validation. These values are a marginal (pseudo) probability that can then be used in a regression model to predict individuals’ probabilities conditional on the observed predictor values. Pseudovalues for the cumulative incidence function at 10 years were regressed on the predictor parameters in a generalised linear model with a complementary log-log link function^52^ ^53^ ^54^ and robust standard errors to account for the non-independence of pseudovalues. The resultant coefficients are statistically similar to those of the Fine-Gray model^52^ ^54^ but computationally less burdensome to obtain, and permit direct modelling of probabilities.

All fitting and evaluation of the Cox and competing risks regression models occurred in each separate imputed dataset, with Rubin’s rules used to pool coefficients and standard errors across all imputations.^55^

### XGBoost and neural network models

The XGBoost and neural network approaches were adapted to handle right censored data in the setting of competing risks by using the jack-knife pseudovalues for the cumulative incidence function at 10 years as a continuous outcome variable. The same predictor parameters as selected for the competing risks regression model were used for the purposes of benchmarking. The XGBoost model used untransformed values for continuous predictors, but these were minimum-maximum scaled (constrained between 0 and 1) for the neural network. We converted categorical variables with more than two levels to dummy variables for both machine learning approaches.

We fit the XGBoost and neural network models to the entire available cohort and used bayesian optimisation^56^ with fivefold cross validation to identify the optimal configuration of hyperparameters to minimise the root mean squared error between observed pseudovalues and model predictions. Fifty iterations of bayesian optimisation were used, with the expected improvement acquisition function.

For the XGBoost model, we used bayesian optimisation to tune the number of boosting rounds, learning rate (eta), tree depth, subsample fraction, regularisation parameters (alpha gamma, and lambda), and column sampling fractions (per tree, per level). We used the squared error regression option as the objective, and the root mean squared error as the evaluation metric.

To permit modelling of higher order interactions in this tabular dataset, we used a feed forward artificial neural network approach with fully connected dense layers: the model architecture comprised an input layer of 26 nodes (ie, number of predictor parameters), rectified linear unit activation functions in each hidden layer, and a single linear activation output node to generate predictions for the pseudovalues of the cumulative incidence function. The Adam optimiser was used,^57^ with the initial learning rate, number of hidden layers, number of nodes in each hidden layer, and number of training epochs tuned using bayesian optimisation. If the loss function had plateaued for three epochs, we halved the learning rate, with early stopping after five epochs if the loss function had not reduced by 0.0001. The loss function was the root mean squared error between observed and predicted pseudovalues due to the continuous nature of the target variable.^58^

After identification of the optimal hyperparameter configurations, we fit the models accordingly to the entirety of the cohort data. We then assessed the performance of these models using the internal-external cross validation strategy—this resembled that for the regression models but with the addition of a hyperparameter tuning component (fig 1). During each iteration of internal-external cross validation, we used bayesian optimisation with fivefold cross validation to identify the optimal hyperparameters for the model fitted to the development data from period 1, which we then tested on the held-out period 2 data. This therefore constituted a form of nested cross validation.^59^

As the XGBoost and neural network models do not constitute a linear set of parameters and do not have standard errors (therefore not able to be pooled using Rubin’s rules), we used a stacked imputation strategy. The 50 imputed datasets were stacked to form a single, long dataset, which enabled us to use the same full data as for the regression models, avoiding suboptimal approaches such as complete case analysis or single imputation. For model evaluation after internal-external cross validation, we used approaches based on Rubin’s rules,^55^ with performance estimates calculated in each separate imputed dataset using the internal-external cross validation generated individual predictions, and then the estimates were pooled.

### Performance evaluation

Predicted risks when using the Cox model can be derived by combining the linear predictor with the baseline hazard function using the equation: predicted event probability=1−St^exp(Xβ)^ where St is the baseline survival function calculated at 10 years, and Xβ is the individual’s linear predictor. For internal-external cross validation, we estimated baseline survival functions separately in each imputation in the period 1 data (continuous predictors centred at the mean, binary predictors set to zero), with results pooled across imputations in accordance with Rubin’s rules.^55^ We estimated the final model’s baseline function similarly but using the full cohort data.

Probabilistic predictions for the competing risks regression model were directly calculated using the following transformation of the linear predictors (Xβ, which included a constant term): predicted event probability=1−exp(−exp(Xβ)).

As the XGBoost and neural network approaches modelled the pseudovalues directly, we handled the generated predictions as probabilities (conditional on the predictor values). As pseudovalues are not restricted to lie between 0 and 1, we clipped the XGBoost and neural network model predictions to be between 0 and 1 to represent predicted probabilities for model evaluation.

Discrimination was assessed using Harrell’s C index,^60^ calculated at 10 years and taking censoring into account—this used inverse probability of censoring weights for competing risks regression, XGBoost, and neural networks given their competing risks formulation.^61^ Calibration was summarised in terms of the calibration slope and calibration-in-the-large.^62^ ^63^ Region level results for these metrics were computed during internal-external cross validation and pooled using random effects meta-analysis^20^ with the Hartung-Knapp-Sidik-Jonkmann method^64^ to provide an estimate of each metric with a 95% confidence interval, and with a 95% prediction interval. The prediction interval estimates the range of model performance on application to a distinct dataset.^20^ We also computed these metrics by ethnicity, 10 year age groups, and cancer stage (I-IV) using the pooled, individual level predictions.

Using the individual level predictions from all models, we generated smoothed calibration plots to assess alignment of observed and predicted risks across the spectrum of predicted risks. We generated these using a running smoother through individual risk predictions, and observed individual pseudovalues^65^ for the Kaplan-Meier failure function (Cox model) or cumulative incidence function (all other models).

Meta-regression following Hartung-Knapp-Sidik-Jonkmann random effects models were used to calculate measures of I^2^ and R^2^ to assess the extent to which inter-regional heterogeneity in discrimination and calibration metrics could be attributable to regional variation in age, BMI (standard deviation thereof), mean deprivation score, and ethnic diversity (percentage of people of non-white ethnicity).^20^ These region level characteristics were estimated using the data from period 2.

We compared the models for clinical utility using decision curve analysis.^66^ This analysis assesses the trade-off between the benefits of true positives (breast cancer deaths) and the potential harms that may arise from false positives across a range of threshold probabilities. Each model was compared using the two default scenarios of treat all or treat none, with the mean model prediction used for each individual across all imputations. This approach implicitly takes into account both discrimination and calibration and also extends model evaluation to consider the ramifications on clinical decision making.^67^ The competing risk of other, non-breast-cancer death was taken into account. Decision curves were plotted overall, and by cancer stage to explore potential utility for all breast cancers.

Predictions generated from the Cox proportional hazards model and other, competing risks approaches have different interpretations, owing to their differential handling of competing events and their modelling of hazard functions with distinct statistical properties.

### Software and code

Data processing, multiple imputation, regression modelling, and evaluation of internal-external cross validation results utilised Stata (version 17). Machine learning modelling was performed in R 4.0.1 (xgboost, keras, and ParBayesianOptimization packages), with an NVIDIA Tesla V100 used for graphical processing unit support. Analysis code is available in repository https://github.com/AshDF91/Breast-cancer-prognosis.

### Patient and public involvement

Two people who survived breast cancer were involved in discussions about the scope of the project, candidate predictors, importance of research questions, and co-creation of lay summaries before submitting the project for approval. This project was also presented at an Oxfordshire based breast cancer support group to obtain qualitative feedback on the study’s aims and face validity or plausibility of candidate predictors, and to discuss the acceptability of clinical risk models to guide stratified breast cancer care.

## Results

### Study cohort and incidence rates

A total of 141 765 women aged between 20 and 97 years at date of breast cancer diagnosis were included in the study. During the entirety of follow-up (median 4.16 (interquartile range 1.76-8.26) years), there were 21 688 breast cancer related deaths and 11 454 deaths from other causes. Restricting to 10 years maximum follow-up from breast cancer diagnosis, 20 367 breast cancer related deaths occurred during a total of 688 564.81 person years. The crude mortality rate was 295.79 per 10 000 person years (95% confidence interval 291.75 to 299.88). Supplementary figure 1 presents ethnic group specific mortality curves. Table 1 shows the baseline characteristics of the cohort overall and separately by decade defined subcohort.

**Table 1:** Summary characteristics of final study cohort overall and separated into temporally distinct subcohorts used in internal-external cross validation. Values are number (column percentage) unless stated otherwise

Variables and categories	Overall study cohort	Subcohorts*	
		Period 1 (1 Jan 2000-31 Dec 2009)	Period 2 (1 Jan 2010-31 Dec 2020)
Total No of individuals	141 765	59 385	82 380
Breast cancer related deaths within 10 years	20 367 (14.37)	7551 (12.72)	8808 (10.69)
Competing events (other cause death) within 10 years	9588 (6.74)	3309 (5.57)	3673 (4.46)
Age at diagnosis:			
Mean (SD)	63.12 (14.31)	63.07 (14.35)	63.15 (14.28)
Median (IQR)	62.62 (52.03-73.64)	62.35 (52.16-74.19)	62.90 (51.94-73.30)
BMI at diagnosis†:			
Mean (SD)	27.18 (5.55)	26.68 (5.27)	27.48 (5.69)
Median (IQR)	26.2 (23.1-30.3)	25.8 (22.9-29.6)	26.5 (23.3-30.7)
Not recorded	22 288 (15.72)	14 066 (23.69)	8222 (9.98)
Mean (SD) Townsend deprivation score	−0.58 (2.95)	−0.59 (2.96)	−0.57 (2.94)
Ethnicity:			
White	92 333 (65.13)	33 473 (56.37)	58 860 (71.45)
Indian	1666 (1.18)	509 (0.86)	1157 (1.40)
Pakistani	866 (0.61)	232 (0.39)	634 (0.77)
Bangladeshi	317 (0.22)	66 (0.11)	251 (0.30)
Other Asian	1019 (0.72)	258 (0.43)	761 (0.92)
Caribbean	1320 (0.93)	411 (0.69)	909 (1.10)
Black African	1096 (0.77)	242 (0.41)	854 (1.04)
Chinese	389 (0.27)	104 (0.18)	285 (0.35)
Other ethnic group (including Arab, mixed race)	1770 (1.25)	396 (0.67)	1374 (1.67)
Not recorded	40 989 (28.91)	23 694 (39.90)	17 295 (20.99)
Smoking status:			
Non-smoker	80 542 (56.81)	31 744 (53.45)	48 798 (59.24)
Former smoker	31 271 (22.06)	10 999 (18.52)	20 272 (24.61)
Light smoker (1-9/day)	14 211 (10.02)	6389 (10.76)	7822 (9.50)
Moderate smoker (10-19/day)	3451 (2.43)	1525 (2.57)	1926 (2.34)
Heavy smoker (≥20/day)	1826 (1.29)	939 (1.58)	887 (1.08)
Not recorded	10 464 (7.38)	7789 (13.12)	2675 (3.25)
Alcohol intake:			
Non-drinker	76 495 (53.96)	29 181 (49.14)	47 314 (57.43)
Trivial (<1 unit/day)	24 711 (17.43)	10 115 (17.03)	14 596 (17.72)
Light (1-2 units/day)	10 123 (7.14)	3761 (6.33)	6362 (7.72)
Moderate (3-6 units/day)	6585 (4.65)	2476 (4.17)	4109 (4.99)
Heavy (7-9 units/day)	276 (0.19)	86 (0.14)	190 (0.23)
Very heavy (>9 units/day)	166 (0.12)	24 (0.04)	142 (0.17)
Not recorded	23 409 (16.51)	13 742 (23.14)	9667 (11.73)
Cancer grade:			
Well differentiated	17 439 (12.30)	8000 (13.47)	9439 (11.46)
Moderately differentiated	54 075 (38.14)	21 868 (36.82)	32 207 (39.10)
Poorly or undifferentiated	34 405 (24.27)	15 153 (25.52)	19 252 (23.37)
Not recorded	35 846 (25.29)	14 364 (24.19)	21 482 (26.08)
Cancer stage:			
I	33 444 (23.59)	10 021 (16.87)	23 423 (28.43)
II	29 731 (20.97)	8258 (13.91)	21 473 (26.07)
III	6358 (4.48)	1410 (2.37)	4948 (6.01)
IV	4057 (2.86)	1046 (1.76)	3011 (3.66)
Not recorded	68 175 (48.09)	38 650 (65.08)	29 525 (35.84)
Route to cancer diagnosis:			
Emergency presentation	2893 (2.04)	1095 (1.84)	1798 (2.18)
Routine referral by GP	7699 (5.43)	3571 (6.01)	4128 (5.01)
Inpatient elective	147 (0.10)	76 (0.13)	71 (0.09)
Other outpatient	1702 (1.20)	841 (1.42)	861 (1.05)
Screening	21 149 (14.92)	6636 (11.17)	14 513 (17.62)
Two week wait	36 438 (25.70)	10 610 (17.87)	25 828 (31.35)
Not recorded	71 737 (50.60)	36 556 (61.56)	35 181 (42.71)
Progesterone receptor status:			
Negative	9297 (6.56)	1366 (2.30)	7931 (9.63)
Positive	20 210 (14.26)	2302 (3.88)	17 908 (21.74)
Not recorded	112 258 (79.19)	55 717 (93.82)	56 541 (68.63)
HER2 status:			
Negative	41 571 (29.32)	3644 (6.14)	37 927 (46.04)
Positive	7239 (5.11)	788 (1.33)	6451 (7.83)
Not recorded	92 955 (65.57)	54 953 (92.54)	38 002 (46.13)
Oestrogen receptor status:			
Negative	7930 (5.59)	1349 (2.27)	6581 (7.99)
Positive	44 696 (31.53)	6682 (11.25)	38 014 (46.14)
Not recorded	89 139 (62.88)	51 354 (86.48)	37 785 (45.87)
All 3 of PR/ER/HER2 status recorded	23 795 (16.78)	2447 (4.12)	21 348 (25.91)
Treatment:			
Mastectomy	40 789 (28.77)	17 939 (30.21)	22 850 (27.74)
Other surgery	69 584 (49.08)	25 740 (43.34)	43 844 (53.22)
Chemotherapy	38 709 (27.31)	12 823 (21.59)	25 886 (31.42)
Radiotherapy	30 275 (21.36)	2696 (4.54)	27 579 (33.48)
Medical history and comorbidities:			
Family history of breast cancer	6315 (4.45)	1916 (3.23)	4399 (5.34)
Type 1 diabetes mellitus	179 (0.13)	82 (0.14)	97 (0.12)
Type 2 diabetes mellitus	11 410 (8.05)	3824 (6.44)	7586 (9.21)
Vasculitis	2308 (1.63)	802 (1.35)	1506 (1.83)
Chronic liver disease or cirrhosis	812 (0.57)	247 (0.42)	565 (0.69)
Chronic kidney disease:			
None/stage 2	131 961 (93.08)	57 410 (96.67)	74 551 (90.50)
Stage 3	8959 (6.32)	1716 (2.89)	7243 (8.79)
Stage 4	498 (0.35)	100 (0.17)	398 (0.48)
Stage 5 (including transplant)	347 (0.24)	159 (0.27)	188 (0.23)
Hypertension	44 259 (31.22)	17 135 (28.85)	27 124 (32.93)
Ischaemic heart disease	8750 (6.17)	3764 (6.34)	4986 (6.05)
Systemic lupus erythematosus	303 (0.21)	101 (0.17)	202 (0.25)
Drug use‡:			
Monoamine oxidase inhibitor	40 (0.03)	24 (0.04)	16 (0.02)
Oral contraceptive	1892 (1.33)	923 (1.55)	969 (1.18)
Other antidepressant	3071 (2.17)	647 (1.09)	2424 (2.94)
RAA antagonist	27 870 (19.66)	9743 (16.41)	18 127 (22.00)
Selective serotonin reuptake inhibitor	12 629 (8.91)	4017 (6.76)	8612 (10.45)
Tricyclic antidepressant	9710 (6.85)	3908 (6.58)	5802 (7.04)
Thiazide	15 769 (11.12)	7791 (13.12)	7978 (9.68)
ACE inhibitor	18 806 (13.27)	7084 (11.93)	11 722 (14.23)
Antipsychotic	2198 (1.55)	1021 (1.72)	1177 (1.43)
β blocker	16 809 (11.86)	7220 (12.16)	9589 (11.64)
HRT (any)	8837 (6.23)	5346 (9.00)	3491 (4.24)
Missing data in ≥1 variable (before imputation)	131 450 (92.72)	58 482 (98.48)	72 967 (88.57)

ACE=angiotensin converting enzyme; BMI=body mass index; GP=general practitioner; HER2 human epidermal growth factor receptor 2; HRT=hormone replacement therapy; IQR=interquartile range; RAA=renin-angiotensin aldosterone; SD=standard deviation.

One alcohol unit is equal to 10 mL or 8 g of pure alcohol.

* For the purposes of internal-external cross validation aiming to mimic wholly temporally distinct datasets and avoid data leakage, follow-up of people entering the cohort during period 1 was truncated at the end of that period if necessary (eg, if they entered in 2002 but died in 2015, they would have been censored at end of period 1); therefore, the numbers of events within 10 years for the subcohorts do not equal the overall summary count.

† Median interval between most recent BMI record and date of breast cancer diagnosis was 597 days (interquartile range 193-1661 days).

‡ Drugs not related to breast cancer treatment refer to use within the six months preceding breast cancer diagnosis.

After the cohort was split by decade of cohort entry and follow-up was truncated for the purposes of internal-external cross validation, 7551 breast cancer related deaths occurred in period 1 during a total of 211 006.95 person years of follow-up (crude mortality rate 357.96 per 10 000 person years (95% confidence interval 349.87 to 366.02)). In the period 2 data, 8808 breast cancer related deaths occurred during a total of 297 066.74 person years of follow-up, with a lower crude mortality rate of 296.50 per 10 000 person years (290.37 to 302.76) observed.

### Cox proportional hazards model

**Fig 2:**
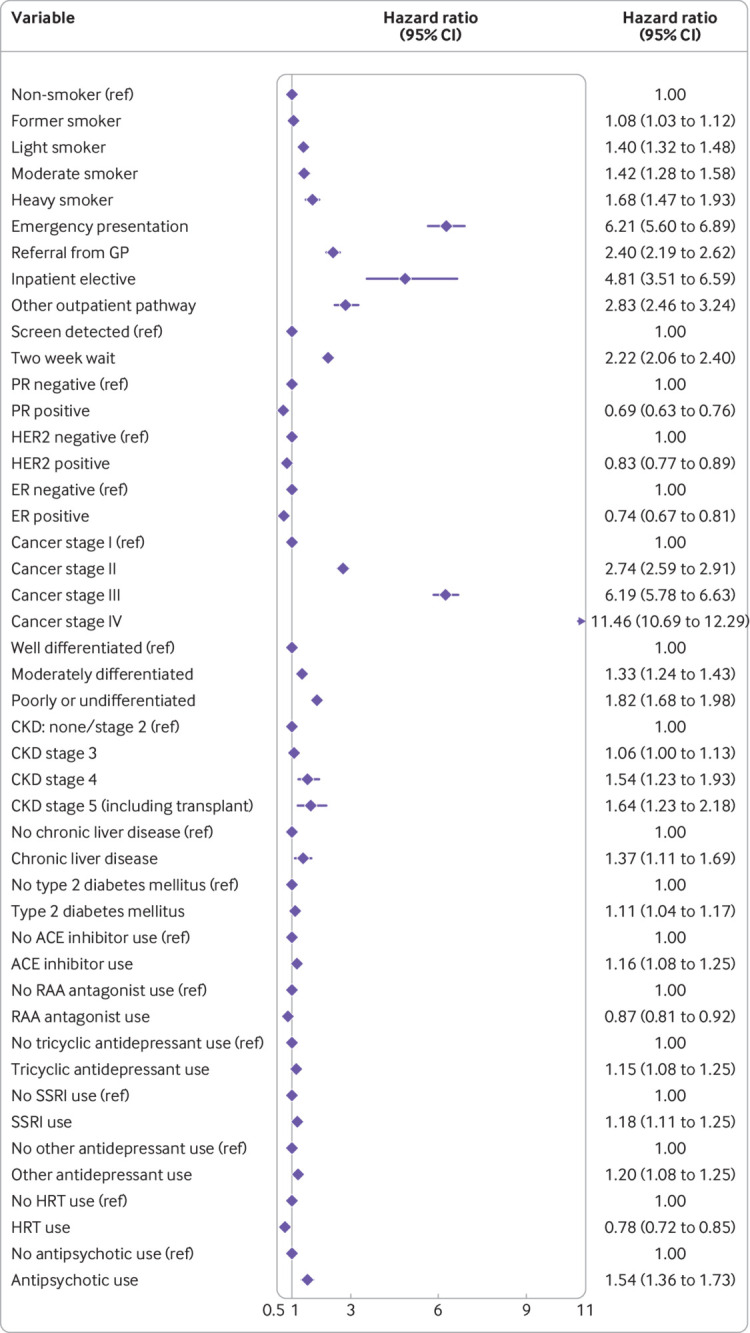
Final Cox proportional hazards model predicting 10 year risk of breast cancer mortality, presented as its exponentiated coefficients (hazard ratios with 95% confidence intervals). Model contains fractional polynomial terms for age (0.5, 2) and body mass index (2, 2), but these are not plotted owing to reasons of scale. Model also includes a baseline survival term (not plotted—the full model as coefficients is presented in the supplementary file). ACE=angiotensin converting enzyme; CI=confidence interval; CKD=chronic kidney disease; ER=oestrogen receptor; GP=general practitioner; HER2= human epidermal growth factor receptor 2; HRT=hormone replacement therapy; PR=progesterone receptor; RAA=renin-angiotensin aldosterone; SSRI=selective serotonin reuptake inhibitor

We selected non-linear fractional polynomial terms for age and BMI (see supplementary figure 2). The final Cox model after predictor selection is presented as exponentiated coefficients in figure 2 for transparency, with the full model detailed in supplementary table 3. Model performance across all ethnic groups is summarised in supplementary table 4: discrimination ranged between a Harrell’s C index of 0.794 (95% confidence interval 0.691 to 0.896) in Bangladeshi women to 0.931 (0.839 to 1.000) in Chinese women, but the low numbers of event counts in smaller ethnic groups (eg, Chinese) meant that overall calibration indices were imprecisely estimated for some.

**Fig 3:**
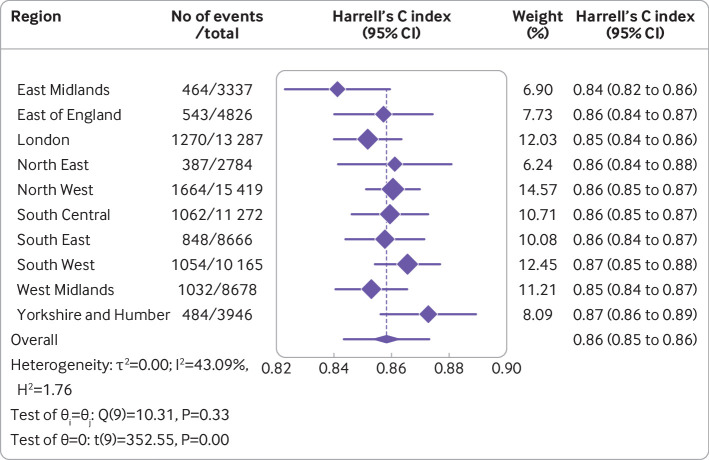
Results from internal-external cross validation of Cox proportional hazards model for Harrell’s C index. Plots display region level performance metric estimates and 95% confidence intervals (diamonds with lines), and an overall pooled estimate obtained using random effects meta-analysis and 95% confidence interval (lowest diamond) and 95% prediction interval (line through lowest diamond). CI=confidence interval

**Fig 4:**
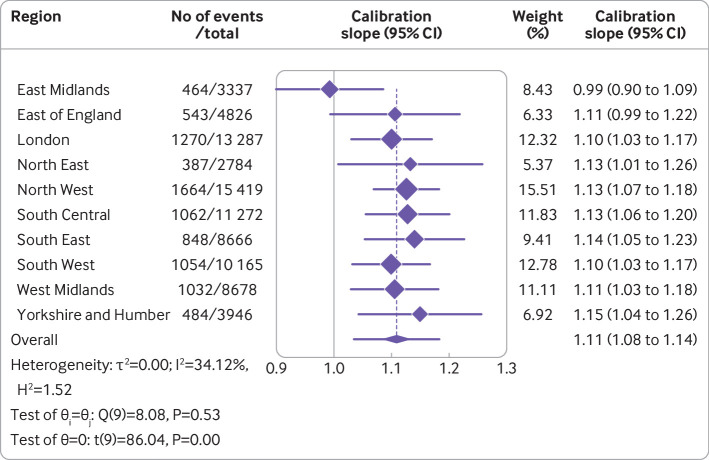
Results from internal-external cross validation of Cox proportional hazards model for calibration slope. Plots display region level performance metric estimates and 95% confidence intervals (diamonds with lines), and an overall pooled estimate obtained using random effects meta-analysis and 95% confidence interval (lowest diamond) and 95% prediction interval (line through lowest diamond). CI=confidence interval

**Fig 5:**
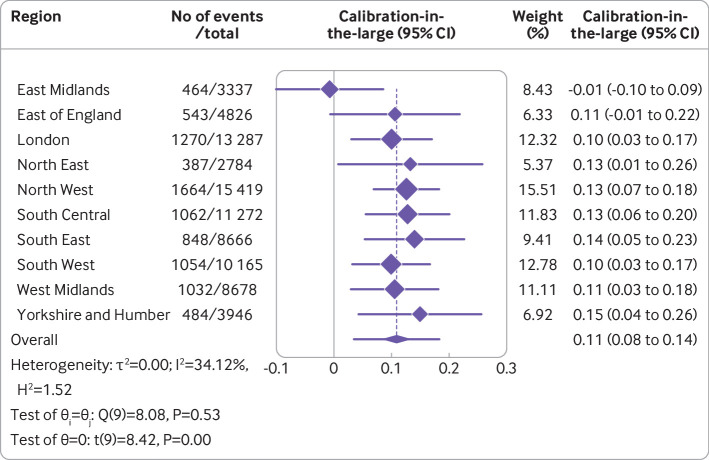
Results from internal-external cross validation of Cox proportional hazards model for calibration-in-the-large. Plots display region level performance metric estimates and 95% confidence intervals (diamonds with lines), and an overall pooled estimate obtained using random effects meta-analysis and 95% confidence interval (lowest diamond) and 95% prediction interval (line through lowest diamond). CI=confidence interval

**Fig 6:**
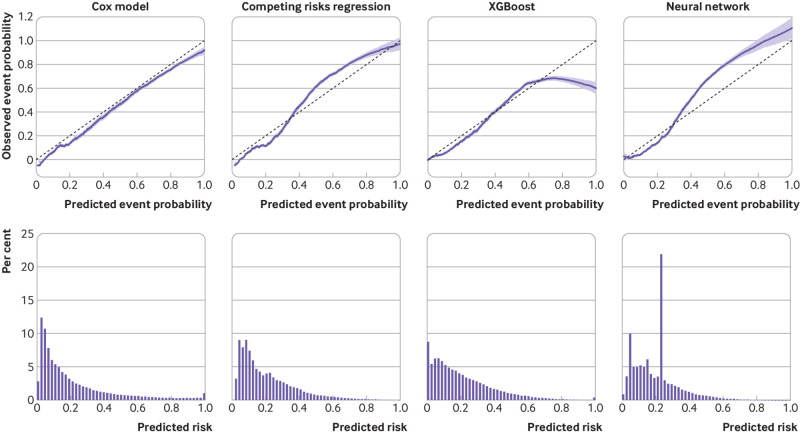
Calibration of the four models tested. Top row shows the alignment between predicted and observed risks for all models with smoothed calibration plots. Bottom row summarises the distribution of predicted risks from each model as histograms

Overall, the Cox model’s random effects meta-analysis pooled estimate for Harrell’s C index was the highest of any model, at 0.858 (95% confidence interval 0.853 to 0.864, 95% prediction interval 0.843 to 0.873). A small degree of miscalibration occurred on summary metrics, with a meta-analysis pooled estimate for the calibration slope of 1.108 (95% confidence interval 1.079 to 1.138, 95% prediction interval 1.034 to 1.182) (table 2). Figure 3, figure 4, and figure 5 show the meta-analysis pooling of performance metrics across regions. Smoothed calibration plots showed generally good alignment of observed and predicted risks across the entire spectrum of predicted risks, albeit with some minor over-prediction (fig 6).

**Table 2:** Summary performance metrics for all four models, estimated using random effects meta-analysis after internal-external cross validation.

Model	Point estimate (95% CI); (95% PI)		
	Harrell’s C index*	Calibration slope	Calibration-in-the-large
Cox proportional hazards	0.858 (0.853 to 0.864); (0.843 to 0.873)	1.108 (1.079 to 1.138); (1.034 to 1.182)	0.108 (0.079 to 0.138); (0.034 to 0.182)
Competing risks	0.849 (0.839 to 0.859); (0.821 to 0.876)	1.160 (1.064 to 1.255); (0.872 to 1.477)	0.160 (0.064 to 0.255); (−0.218 to 0.447)
XGBoost	0.821 (0.813 to 0.828); (0.805 to 0.837)	1.084 (1.003 to 1.165); (0.842 to 1.326)	0.084 (0.003 to 0.165); (−0.158 to 0.326)
Neural network	0.847 (0.835 to 0.858); (0.816 to 0.878)	1.037 (0.910 to 1.165); (0.624 to 1.451)	0.037 (−0.090 to 0.165); (−0.376 to 0.451)

CI=confidence interval; PI=prediction interval.

* Inverse probability of censoring weights was used to estimate Harrell’s C index for competing risks, XGBoost, and neural network models.

Regional differences in the Harrell’s C index were relatively slight. None of the inter-region heterogeneity observed for discrimination (I^2^=53.14%) and calibration (I^2^=42.35%) appeared to be attributable to regional variation in any of the sociodemographic factors examined (table 3). The model discriminated well across cancer stages, but discriminative capability decreased with increasing stage; moderate variation was observed in calibration across cancer stage groups (supplementary table 9).

**Table 3:** Random effects meta-regression of relative contributions of regional variation in age, body mass index, deprivation, and non-white ethnicity on inter-regional differences in performance metrics after internal-external cross validation

Performance metric and variables	Cox model			Competing risks model			XGBoost			Neural network	
	I^2^ (%)	R^2^ (%)		I^2^ (%)	R^2^ (%)		I^2^ (%)	R^2^ (%)		I^2^ (%)	R^2^ (%)
**Harrell’s C index**											
Age (SD)	43.06	0.00		41.45	9.54		17.64	0.00		43.68	8.05
BMI (SD)	45.72	0.00		47.96	0.00		16.78	0.00		49.16	0.00
Mean Townsend deprivation score	44.28	0.00		46.25	0.00		15.87	3.34		48.29	0.00
Non-white ethnicity (%)	45.47	0.00		42.34	11.02		17.65	0.00		25.67	61.11
All 4 variables	53.14	0.00		41.33	8.63		16.43	0.88		30.40	48.25
**Calibration slope**											
Age (SD)	35.45	0.00		70.04	26.09		66.44	23.58		89.29	28.95
BMI (SD)	35.93	0.00		77.14	0.00		74.72	0.00		93.20	0.00
Mean Townsend deprivation score	35.90	0.00		73.63	12.05		72.33	0.00		93.16	0.00
Non-white ethnicity (%)	34.21	0.00		58.49	55.77		56.06	51.91		87.07	42.31
All 4 variables	42.35	0.00		56.68	60.08		50.18	61.97		67.78	80.06

BMI=body mass index; SD=standard deviation.

I^2^=residual heterogeneity; R^2^=amount of residual heterogeneity accounted for.

### Competing risks regression

**Fig 7:**
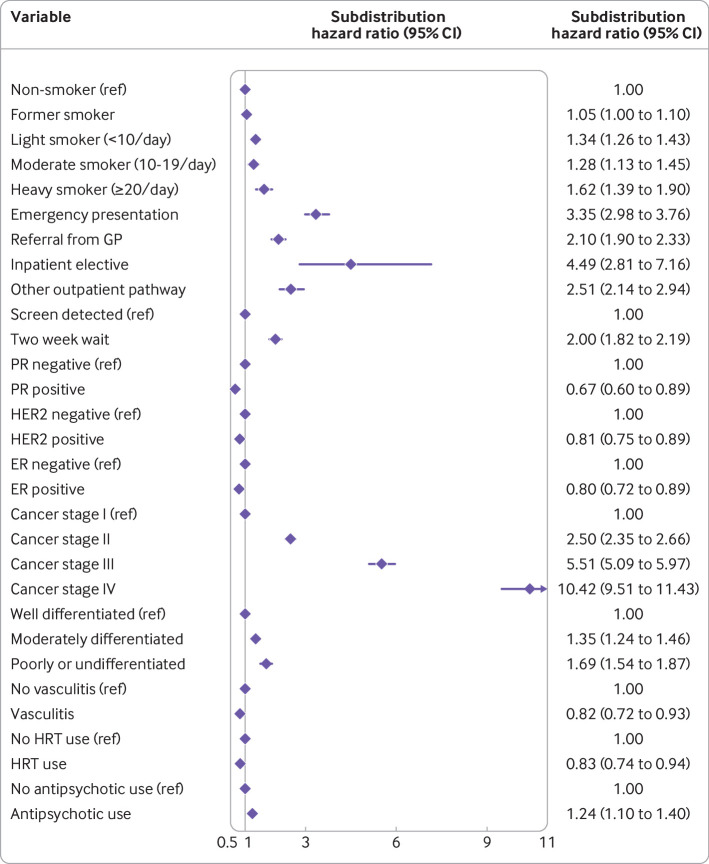
Final competing risks regression model predicting 10 year risk of breast cancer mortality, presented as its exponentiated coefficients (subdistribution hazard ratios with 95% confidence intervals). Model contains fractional polynomial terms for age (1, 2) and body mass index (2, 2), but these are not plotted owing to reasons of scale. Model also includes an intercept term (not plotted—see supplementary file for full model as coefficients). CI=confidence interval; ER=oestrogen receptor; GP=general practitioner; HER2=human epidermal growth factor receptor 2; HRT=hormone replacement therapy; PR=progesterone receptor

Similar fractional polynomial terms were selected for age and BMI in the competing risks regression model (see supplementary figure 2), and predictor selection yielded a model with fewer predictors than the Cox model. The competing risks regression model is presented as exponentiated coefficients in figure 7, with the full model (including constant term) detailed in supplementary table 5. Ethnic group specific discrimination and overall calibration metrics are detailed in supplementary table 4—the model generally performed well across ethnic groups, with similar discrimination, but there was some overt miscalibration on summary metrics—although some metrics were estimated imprecisely owing to small event counts in some ethnic groups.

The random effects meta-analysis pooled Harrell’s C index was 0.849 (95% confidence interval 0.839 to 0.859, 95% prediction interval 0.821 to 0.876). Some evidence suggested systematic miscalibration overall—that is, a pooled calibration slope of 1.160 (95% confidence interval 1.064 to 1.255, 95% prediction interval 0.872 to 1.447). Smoothed calibration plots showed underestimation of risk at the highest predicted values (eg, predicted risk >40%, fig 6). Supplementary figure 3 displays regional performance metrics.

An estimated 41.33% of the regional variation in the Harrell’s C index for the competing risks regression model was attributable to inter-regional case mix (table 3); ethnic diversity was the leading sociodemographic factor associated therewith (table 3). For calibration, the I^2^ from the full meta-regression model was 56.68%, with regional variation in age, deprivation, and ethnic diversity associated therewith. Similar to the Cox model, discrimination tended to decrease with increasing cancer stage (supplementary table 9).

### XGBoost

Table 4 summarises the selected hyperparameter configuration for the final XGBoost model. The discrimination of this model appeared acceptable overall,^68^ albeit lower than for both regression models (table 2; supplementary figure 4), with a meta-analysis pooled Harrell’s C index of 0.821 (95% confidence interval 0.813 to 0.828, 95% prediction interval 0.805 to 0.837). Pooled calibration metrics suggested some mild systemic miscalibration—for example, the meta-analysis pooled calibration slope was 1.084 (95% confidence interval 1.003 to 1.165, 95% prediction interval 0.842 to 1.326). Calibration plots showed miscalibration across much of the predicted risk spectrum (fig 6), with overestimation in those with predicted risks <0.4 (most of the individuals) before mixed underestimation and overestimation in the patients at highest risk. Discrimination and calibration were poor for stage IV tumours (see supplementary table 9). Regarding regional variation in performance metrics as a result of differences between regions, most of the variation in calibration was attributable to ethnic diversity, followed by regional differences in age (table 3).

**Table 4:** Description of machine learning model architectures and hyperparameters tuning performed

Model, basic architecture, and hyperparameters tuned	Range explored during tuning	Final selected value after tuning
**XGBoost**		
Tree based booster with GPU_hist; gradient based subsampling; RMSE evaluation metric	1-6	6
Maximum tree depth:		
Learning rate (eta)	0.0001-0.1	0.073
Subsampling proportion	0.1-0.5	0.1
No of boosting rounds	1-500	251
Alpha (regularisation)	0-20	18
Gamma (regularisation)	0-20	0
Lambda (regularisation)	0-20	3
Column sampling by tree	0.1-0.8	0.501
Column sampling by level	0.1-0.8	0.518
**Neural network**		
Feed forward ANN with fully connected layers; 26 input nodes (No of predictors); ReLU activation functions in hidden layers; Adam optimiser; single output node with linear activation; RMSE loss function; batch size 1024:		
No of hidden layers	1-5	2
No of nodes in each hidden layer	26-50	30
No of epochs	1-50	32
Initial learning rate	0.001-0.1	0.032

ANN=artificial neural network; ReLU=rectified linear unit; RMSE=root mean squared error.

The continuous outcome variables for both models were the jack-knife pseudovalues for the cumulative incidence function for breast cancer related mortality at 10 years. The final neural network model had a total of 1771 parameters (all trainable).

### Neural network

Table 4 summarises the selected hyperparameter configuration for the final neural network. This model performed better than XGBoost for overall discrimination—the meta-analysis pooled Harrell’s C index was 0.847 (95% confidence interval 0.835 to 0.858, 95% prediction interval 0.816 to 0.878, table 2 and supplementary figure 5). Post-internal-external cross validation pooled estimates of summary calibration metrics suggested no systemic miscalibration overall, such as a calibration slope of 1.037 (95% confidence interval 0.910 to 1.165), but heterogeneity was more noticeable across region, manifesting in the wide 95% prediction interval (slope: 0.624 to 1.451), and smoothed calibration plots showed a complex pattern of miscalibration (fig 6). Meta-regression estimated that the leading factor associated with inter-regional variation in discrimination and calibration metrics was regional differences in ethnic diversity (table 3).

### Stage specific performance and decision curve analysis

**Fig 8:**
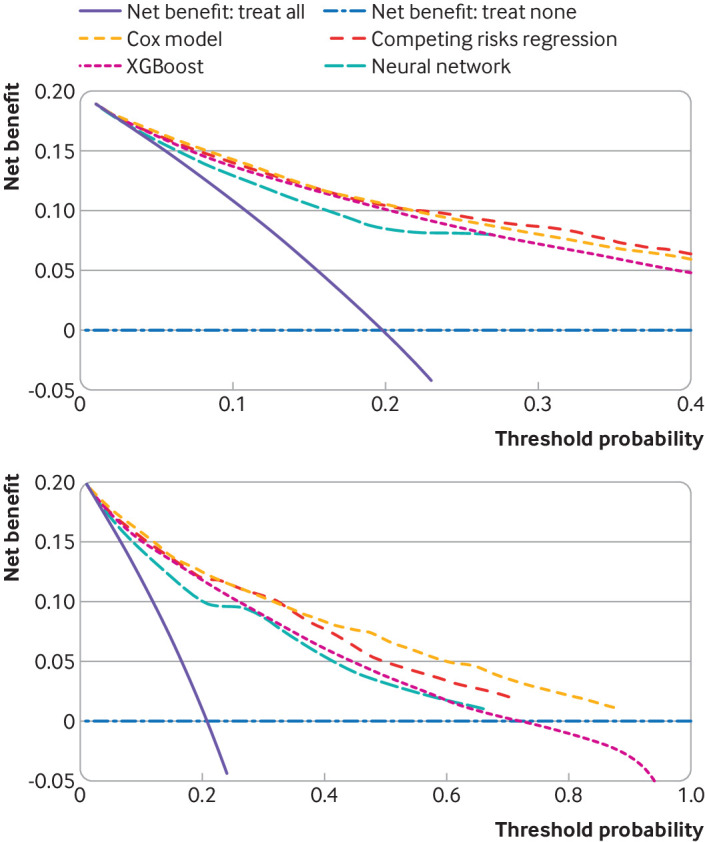
Decision curves to assess clinical utility (net benefit) of using each model. Top plot accounts for the competing risk of other cause mortality. Bottom plot does not account for competing risks

Both the XGBoost and neural network approaches showed erratic calibration across cancer stage groups, especially major miscalibration in stage III and IV tumours, such as a slope for the neural network of 0.126 (95% confidence interval 0.005 to 0.247) in stage IV tumours (see supplementary table 9). Overall decision curves showed that when accounting for competing risks, net benefit was generally better for the regression models, and the neural network had lowest clinical utility; when not accounting for competing risks, the regression models had higher net benefit across the threshold probabilities examined (fig 8). Lastly, the clinical utility of the machine learning models was variable across tumour stages, such as null or negative net benefit compared with the scenarios of treat all for stage IV tumours (see supplementary figure 6).

### Clinical scenarios and risk predictions

Table 5 illustrates the predictions obtained using the Cox and competing risks regression models for different sample scenarios. When relevant, these are compared with predictions for the same clinical scenarios from PREDICT Breast and the Adjutorium model (obtained using their web calculators: https://breast.predict.nhs.uk/ and https://adjutorium-breastcancer.herokuapp.com).

**Table 5:** Risk predictions from Cox and competing risks regression models developed in this study for illustrative clinical scenarios, compared where relevant with PREDICT and Adjutorium*

Clinical scenario	Present study		PREDICT Breast	Adjutorium
	Cox model	Competing risks model		
65 year old woman, BMI 30, postmenopausal, ER+, PR+, HER2−, Ki67 <10%, tumour size 9 mm, well differentiated, screen detected invasive cancer, 2 positive nodes, stage II, family history of breast cancer, light smoker, planned for surgery	6.05% risk of breast cancer mortality within 10 years	8.69% risk of breast cancer mortality within 10 years	79% overall survival at 10 years	7% risk of breast cancer mortality within 10 years (82% overall survival)
75 year old woman, BMI 40, postmenopausal, ER+, PR+, HER2+, tumour size 11 mm, well differentiated, referred on 2 week wait pathway by general practitioner after finding breast lump, 4 positive nodes, stage II, Ki67 <10%, non-smoker, planned for surgery	12.99% risk of breast cancer mortality within 10 years	5.73% risk of breast cancer mortality within 10 years	70% overall survival at 10 years	14% risk of breast cancer mortality within 10 years (60% overall survival)
40 year old woman, BMI 33, triple negative, stage III, moderately differentiated, referred on 2 week wait pathway for breast symptoms, heavy smoker	73.05% risk of breast cancer mortality within 10 years	77.92% risk of breast cancer mortality within 10 years	NA	NA

+ =positive; −=negative; BMI=body mass index; ER=oestrogen receptor; HER2=human epidermal growth factor receptor 2; Ki67=nuclear antigen (marker of cell proliferation); NA=not applicable as this model is not intended for use in this patient; PR=progesterone receptor.

* Web calculators accessed 18 July 2022.

## Discussion

This study developed and evaluated four models to estimate 10 year risk of breast cancer death after diagnosis of invasive breast cancer of any stage. Although the regression approaches yielded models that discriminated well and were associated with favourable net benefit overall, the machine learning approaches yielded models that performed less uniformly. For example, the XGBoost and neural network models were associated with negative net benefit at some thresholds in stage I tumours, were miscalibrated in stage III and IV tumours, and exhibited complex miscalibration across the spectrum of predicted risks.

### Strengths and limitations of this study

Study strengths include the use of linked primary and secondary healthcare datasets for case ascertainment, identification of clinical diagnoses using accurately coded data, and avoidance of selection and recall biases. Use of centralised national mortality registries was beneficial for ascertainment of the endpoint and competing events. Our methodology enabled the adaptation of machine learning models to handle time-to-event data with competing risks and inclusion of multiple imputation so that all models benefitted from maximal available information, and the internal-external cross validation framework^28^ permitted robust assessment of model performance and heterogeneity across time, place, and population groups.

Limitations include no consideration of genetic data such as presence of high risk mutations or multigene or multigenomics data, or breast density, which could have offered additional predictive utility.^69^ ^70^ ^71^ Model development depended on using variables that are routinely collected in primary care, Hospital Episodes Statistics data, and the national cancer registry. Reliance on clinical coding for variables such as family history of breast cancer may be skewed towards those with more notable pedigrees; furthermore, as those without recorded positive family history were assumed to have none, misclassification might have occurred. Misclassification bias could also occur with prescriptions data because not all drugs are dispensed by a pharmacist or taken by the individual. Importantly, no coefficient in any model has a causal interpretation, and further work would be required to assess the relevance of altering factors such the management of type 2 diabetes,^72^ or accounting for treatment drop-in^73^ ^74^ ^75^ after diagnosis if a causal prediction component was desired, or a counterfactual treatment selection function.

Another approach to model evaluation is bootstrapping, which allows estimation of optimism during model fitting, and calculation of bias corrected performance metrics. The best approach for combining bootstrapping with multiply imputed data may be to impute each individual bootstrap sample^76^—this would have been computationally intractable for this study, particularly for the machine learning models, which would have additional overhead of hyperparameter tuning in addition to imputation in each resample.

### Comparisons with other studies

In a previous systematic review, the authors identified 58 papers concerning prognostic models for breast cancer.^24^ While the Nottingham Prognostic Index retained its performance in several external evaluations, some other models have performed less well on application to external datasets, such as in patients at the highest ranges of age and risk, which underscores the need for robust assessment of model performance.^24^ The PREDICT Breast model is endorsed by the American Joint Committee on Cancer, and the model is widely used around the world in clinical decision making about adjuvant chemotherapy—however, external evaluations suggest that PREDICT performs less well in older women, and in other subgroups such as women with large, oestrogen negative cancers,^77^ reinforcing the need for consideration of relevant subgroup performance in the prediction of breast cancer outcomes. More relevant to the present study is the lack of a reliable clinical prediction model for our outcome of interest, applicable across all women with breast cancer. The only model^27^ for this clinical scenario found to be at low risk of bias in a published systematic review^25^ likely had too small a sample size to fit a prediction model, unnecessarily dichotomised predictor variables, and the final model was selected after developing more than 35 000 other models.

A recent study to develop and externally validate the Adjutorium model based on automated machine learning focused on treatment recommendation in the postsurgical (adjuvant) setting, reporting that the model derived from the AutoPrognosis approach was superior for discrimination than a Cox proportional hazards model fitted to the same data.^9^ It is, however, notable that the comparison was the full complexity and flexibility of an ensemble machine learning model against a Cox model with no interactions and a simple, single, predetermined (ie, not data driven) non-linearity with a quadratic term for age. Our approach considered up to two fractional polynomial powers, which may be able to capture more complex non-linearities if present, considered regression model interactions, sought to identify optimal models for prognostication more generally in patients with breast cancer, and explored several performance metrics from the perspective of geographical and temporal transportability.

Previous studies discuss the adaptation of machine learning models to handle time-to-event data using jack-knife pseudovalues,^58^ ^78^ ^79^ but in our study we performed comparative evaluation of the discrimination, calibration, and clinical utility of these models in large datasets. In the current study we also report a variation of an XGBoost algorithm that can handle competing risks. Recent developments in machine learning modelling approaches include DeepSurv or DeepHit as adaptations of time-to-event modelling,^80^ whereas our approach directly modelled risk probabilities. Extensions include complex model ensembles such as the Survival Quilts approach, where machine learning models are temporally joined to estimate risks over timespans.^8^ However, we opted for simpler model architectures that are arguably more transparent than meta-model ensembles, and they are conducive to a more robust validation strategy within a set computational budget. Furthermore, the added benefit of using complex models to obtain (at best) modest yields in an overall performance metric such as a C index, as has been shown in recent healthcare machine learning papers,^81^ is debatable. Although our goal was to develop robust models that effectively prognosticate for all breast cancers, a comparative evaluation of the PREDICT and Adjutorium models could have been an interesting analysis in the early breast cancer group treated with surgery. This was not, however, possible owing to systematically missing covariates in our dataset.

It should be noted that no single approach is always optimal for any modelling task—more flexible methods could have better performance in other scenarios if features and risk associations in the given data are complex. Results from this specific modelling scenario on relative performance of different approaches may not hold across all other prediction studies, mandating careful consideration of methodology should more than one modelling approach be used.

### Implications of results and future research

This study shows how comparative evaluation of modelling techniques within an internal-external validation framework in large, clustered healthcare datasets may provide insight into relative strengths of different strategies for clinical prognostication. Regardless of the flexibility of modelling strategy used, all clinical prediction algorithms should be extensively evaluated and stress tested: showing that a model works overall is subservient to understanding if, where, and how a clinical prediction model will break down.

### Conclusions

In this low dimensional clinical setting, a Cox model and a competing risks regression model provided accurate estimation of breast cancer mortality risks in the general population of people of female sex with breast cancer. Subject to independent external validation and cost effectiveness and impact assessments, the two models could have clinical utility, such as informing stratified follow-up regimens.^4^ It is possible that the most robust clinical applications could be attained with future integration of multimodal data, such as genomic markers.^82^ ^83^ Implementation of the models in another clinical dataset, such as one based on electronic healthcare records, may be possible. This should follow local validation and potential recalibration—discrimination could be similar on application to a different system, but calibration may be affected by local variations in rates or treatment practices. The models do not include predictors such as ethnicity or race or deprivation score, which would otherwise need adaptation or scaling to match local metrics. The included predictors would be available to clinical teams caring for women with breast cancer after initial diagnostic investigations and would need to be aligned with local coding systems (eg, SNOMED).

**What is already known on this topic**

Clinical prediction models are used for adjuvant treatment selection in early stage, surgically treated breast cancer, but models that can prognosticate well in breast cancer of any stage could more widely inform risk based follow-up strategies and support prognosis counselling or clinical trial recruitment
Most breast cancer prediction models have methodological limitations, are poorly reported, and are at high risk of bias—the only model deemed at low risk of bias in a recent systematic review for the unselected population of women with breast cancer likely had too small a sample size for development, and uncertain transportability
Considerable interest in machine learning for clinical prediction exists, but there has been criticism of model explainability, transparency, robustness of evaluation, fairness of comparisons, and risks of algorithmic bias

**What this study adds**

A Cox proportional hazards and a competing risks regression model may have utility for informing risk stratified clinical strategies for unselected women with breast cancer—these approaches were superior to the models developed using machine learning methods
An internal-external cross validation framework can be used to identify best performing modelling strategies by assessing model performance, performance heterogeneity, and transportability
Adapting machine learning models to handle censored data in the setting of competing risks can be achieved using a pseudovalues based approach

## Data Availability

To guarantee the confidentiality of personal and health information only the authors have had access to the data during the study in accordance with the relevant licence agreements. Access to the QResearch database is managed, overseen, and approved by the QResearch Scientific Committee.

## Web extra

Extra material supplied by authors

## Notes

Contributors: JH-C and GSC are joint senior authors. AKC, GSC, and JHC led the study conceptualisation and statistical analysis plans. AKC drafted the first version of the study protocol, which all co-authors reviewed, provided input into, and approved. AKC undertook the data specification (reviewed by JHC), data curation and preparation, and analyses, and wrote the first draft of the paper. All other authors reviewed the manuscript, provided critical feedback, and approved the final version. AKC and JHC had full access to the study data. Advanced statistical input was from GSC and JHC. DD and SL provided clinical (oncological) input. AKC has final responsibility for submission and is the guarantor. The corresponding author attests that all listed authors meet authorship criteria and no others meeting the criteria have been omitted.

The lead author (AKC) affirms that the manuscript is an honest, accurate, and transparent account of the study being reported; that no important aspects of the study have been omitted; and that any discrepancies from the study as planned (and, if relevant, registered) have been explained.

Dissemination to participants and related patient and public committees: We plan to invite the patient and public involvement volunteers to use the results to co-create lay summaries and other public facing materials for the purposes of dissemination.

Provenance and peer review: Not commissioned; externally peer reviewed.
