# Supplementary material for: Development and internal-external validation of statistical and machine learning models for breast cancer prognostication: cohort study
Source: BMJ. 2023 May 10;381:e073800. doi: 10.1136/bmj-2022-073800 (PMC10170264; doi:10.1136/bmj-2022-073800)
Supplement: Supplementary file 1 — Supplementary information: additional tables 1-10 [file clia073800.ww1.pdf]

Supplementary Table 1. Methodological issues with the only other model for the same intended use rated as "low risk of bias" in a recent systematic review.

Author & Title (DOI)	Study population	Endpoint(s)	Methodological issue(s)
Paredes-Aracil, et al. A scoring system to predict breast cancer mortality at 5 and 10 years<br><br>(https://www.nature.com/articles/s41598-017-00536-7)	287 women diagnosed with breast cancer at the Elda Health Department, Spain (2003-2006)	5- and 10-year breast cancer mortality	*1) Over 35,000 models examined* – unsure how this was accounted for during model selection. As such, 'testimation bias' or 'data dredging' may be a concern<br><br>*2) Dichotomisation of predictors (age) in final score*<br><br>*3) Insufficient sample size for model development*<br><br>Sample size calculation were based on events-per-variable (study published prior to best practice guidance on estimating minimum sample size).<br><br>We applied the methods of Riley, et al. to this study sample to estimate minimum sample size to develop a clinical prediction model with a time-to-event framework. Based on following parameters obtained from the paper:<br><br>Breast cancer mortality rate 222/10,000 person years = annual rate 0.0222<br>Number of predictor parameters in final model = 9<br>Mean follow-up 8.6 years<br>Prediction horizons: 5 and 10 (years)<br>15% of maximum permitted Cox-Snell R^2 permitted in this scenario = 0.09560669<br>Minimum sample size for both models = **802** (EPP=17.01) versus **287** used in study<br><br>*R code:*<br>*test5 <- pmsampsize(type="s", rsquared = 0.09560669, parameters = 9, timepoint = 5, meanfup = 8.6, rate=0.0222)*<br><br>*test10 <- pmsampsize(type="s", rsquared = 0.09560669, parameters = 9, timepoint = 10, meanfup = 8.6, rate=0.0222)*

Of the 27 models found to be at overall low risk of bias in the systematic review of Hueting, et al. J Clin Epidemiol 2022 doi:https://doi.org/10.1016/j.jclinepi.2022.10.016, 10 predicted survival/mortality outcomes.

Nine of these 10 are not considered above as their intended use population does not align with the current study (i.e. any woman diagnosed with breast cancer):

Candido Do Reis, et al. An updated PREDICT breast cancer prognostication and treatment benefit prediction model with independent validation. doi:10.1186/s13058-017-0852-3. *Only considers women that have undergone surgery*

Wen, et al. Development and validation of a nomogram for predicting survival on the base of modified lymph node ratio in breast cancer patients. doi:10.1016/j.breast.2017.01.017. *Only examined women that have undergone surgery (axillary lymph node clearance). Their exclusion criteria included women diagnosed with metastatic disease.*

Fontein, et al. Dynamic prediction in breast cancer: proving the feasibility in clinical practice using the TEAM trial. doi:10.1093/annonc/mdv146. *Only included post-menopausal, endocrine-sensitive early stage breast cancers.*

Wishart, et al. PREDICT Plus: development and validation of a prognostic model for early breast cancer that includes HER2. doi:10.1038/bjc.2012.338. *Only early stage breast cancers in women that underwent surgery*.

Lee, et al. A nomogram to predict survival time in women starting first-line chemotherapy for advanced breast cancer. doi:10.1007/s10549-011-1471-9. *Only women with advanced disease treated specifically with chemotherapy*.

Ward, et al. Bridging the Age Gap: a prognostic model that predicts survival and aids in primary treatment decisions for older women with oestrogen receptor-positive early breast cancer. https://doi.org/10.1002/bjs.11748. *Developed and evaluated 4 models – all of which have an intended population of women aged 70 years and above, with ER+ breast cancer.*

**Data source/combination**	**Number of cases identified (% of total)**
Case coded in primary care record	118,685 (83.72%)
Case coded in primary care record or HES	136,369 (96.19%)
Case coded in primary care or cancer registry	137,324 (96.87%)
All women in final study cohort	141,765 (100%)

Supplementary Table 2. Ascertainment of breast cancer diagnoses from different combinations of the linked source datasets. The combinations are those generated during processing for data extracts from QResearch. HES = Hospital Episode Statistics.

Supplementary Table 3. Full Cox model – coefficients and baseline survival.

Parameter	Description	Coefficient
**Age at diagnosis (1st FP term)**	X^0.5 - 2.510345299<br>X = age/10	-2.4200838
**Age at diagnosis (2nd FP term)**	X^2 - 39.71310575<br>X = age/10	0.06170782
**BMI at diagnosis (1st FP term)**	X^-2 - 0.1353749689<br>X = BMI/10	3.5626427
**BMI at diagnosis (2nd FP term)**	X^-2*ln(X) - 0.1353551232<br>X = BMI/10	-8.2391665
**Smoking status**	Non-smoker (reference)	0
	Ex-smoker	0.07487781
	Light smoker	0.33338149
	Moderate smoker	0.35168628
	Heavy smoker	0.51960507
**Route to breast cancer diagnosis**	Emergency presentation	1.8266128
	GP referral	0.87391787
	Inpatient elective	1.5714949
	Other outpatient	1.0395208
	Screening (reference)	0
	Two-week wait	0.79912031
**Progesterone receptor status**	Negative (reference)	0
	Positive	-0.36406436
**HER2 status**	Negative (reference)	0
	Positive	-0.18971053
**Oestrogen receptor status**	Negative (reference)	0
	Positive	-0.30338542
**Cancer stage at diagnosis**	Stage 1 (reference)	0
	Stage 2	1.0091567
	Stage 3	1.8234394
	Stage 4	2.4389817
**Cancer grade**	Well differentiated (reference)	0
	Moderately differentiated	0.28412054
	Poorly or undifferentiated	0.59900683
**Chronic kidney disease**	None/Stage 2 (reference)	0
	Stage 3	0.06287756
	Stage 4	0.43083109
	Stage 5 (inc. transplant)	0.49398542
**Chronic liver disease**	None (reference)	0
	Yes	0.316256
**Type 2 diabetes mellitus**	No (reference)	0
	Yes	0.10158724
**ACE inhibitor use**	No (reference)	0
(within 6 months prior)	Yes	0.14908664
**Renin angiotensin axis inhibitor use**	No (reference)	0

(within 6 months prior)	Yes	-0.14476126
**Tricyclic antidepressant use**	No (reference)	0
(within 6 months prior)	Yes	0.13668533
**Selective serotonin reuptake inhibitor use**	No (reference)	0
(within 6 months prior)	Yes	0.16313084
**Other antidepressant use**	No (reference)	0
(within 6 months prior)	Yes	0.18472118
**HRT use**	No (reference)	0
(within 6 months prior)	Yes	-0.285108
**Anti-psychotic use**	No (reference)	0
(within 6 months prior)	Yes	0.43001608
**Baseline survival function at 10 years**		0.9592283

		Cox proportional hazards model			Competing risks regression model		
Ethnic group	Events / denominator	Harrell's C	Calibration slope	Calibration-in-the-large	Harrell's C*	Calibration slope	Calibration-in-the-large
White	5,980 / 58,860	0.860 (0.855 to 0.864)	1.106 (1.077 to 1.136)	0.106 (0.077 to 0.136)	0.848 (0.840 to 0.856)	1.183 (1.128 to 1.238)	0.184 (0.128 to 0.238)
Indian	92 / 1,157	0.849 (0.810 to 0.888)	1.150 (0.960 to 1.340)	0.150 (-0.040 to 0.340)	0.843 (0.780 to 0.906)	1.367 (1.029 to 1.705)	0.367 (0.029 to 0.705)
Pakistani	57 / 634	0.845 (0.790 to 0.899)	1.277 (0.935 to 1.618)	0.277 (-0.065 to 0.618)	0.812 (0.720 to 0.903)	1.230 (0.821 to 1.639)	0.230 (-0.179 to 0.639)
Bangladeshi	24 / 251	0.794 (0.691 to 0.896)	1.057 (0.571 to 1.543)	0.057 (-0.429 to 0.543)	0.777 (0.645 to 0.909)	1.666 (0.939 to 2.394)	0.666 (-0.061 to 1.394)
Other Asian	43 / 761	0.818 (0.752 to 0.884)	1.105 (0.790 to 1.420)	0.105 (-0.210 to 0.420)	0.849 (0.757 to 0.941)	1.548 (1.104 to 1.993)	0.548 (0.104 to 0.993)
Black Caribbean	126 / 909	0.822 (0.784 to 0.860)	1.097 (0.895 to 1.299)	0.097 (-0.105 to 0.299)	0.853 (0.801 to 0.904)	1.188 (0.901 to 1.475)	0.188 (-0.099 to 0.475)
Black African	99 / 854	0.831 (0.788 to 0.874)	0.996 (0.806 to 1.186)	-0.004 (-0.194 to 0.186)	0.822 (0.760 to 0.883)	1.106 (0.783 to 1.429)	0.106 (-0.217 to 0.429)
Chinese	9 / 285	0.931 (0.839 to 1.000)	1.900 (0.984 to 2.817)	0.900 (-0.016 to 1.817)	0.916 (0.802 to 1.000)	1.940 (1.115 to 2.764)	0.940 (0.115 to 1.764)
Other ethnic group	88 / 1,374	0.821 (0.772 to 0.869)	1.124 (0.910 to 1.339)	0.124 (-0.090 to 0.339)	0.834 (0.769 to 0.898)	1.667 (1.301 to 2.034)	0.667 (0.301 to 1.034)

Supplementary Table 4. Ethnic group-specific regression model performance metrics (with 95% confidence intervals) estimated after internal-external cross-validation in the data from period 2 (2010-2020). Event and denominator counts for each ethnic group are from the 'complete case' data for reference, but performance metrics were calculated using the multiply imputed datasets. Harrell's C for the competing risks regression model was weighted by the inverse probability of censoring.

Supplementary Table 5. Full competing risks regression model – coefficients and constant term.

Parameter	Description	Coefficient
**Age at diagnosis (1st FP term)**	X – 6.301833523<br>X = age/10	-0.33729257
**Age at diagnosis (2nd FP term)**	X^2 - 39.71310575<br>X = age/10	0.04244611
**BMI (1st FP term)**	X^-2 - 0.1353749689<br>X = BMI/10	2.2932369
**BMI (2nd FP term)**	X^-2*ln(X) - 0.1353551232<br>X = BMI/10	-5.9806931
**Smoking status**	Non-smoker (reference)	0
	Ex-smoker	0.04905292
	Light smoker	0.29232912
	Moderate smoker	0.24918172
	Heavy smoker	0.48453944
**Route to breast cancer diagnosis**	Emergency presentation	1.208285
	GP referral	0.74362281
	Inpatient elective	1.5009607
	Other outpatient	0.9191697
	Screening (reference)	0
	Two-week wait	0.6927579
**Progesterone receptor status**	Negative (reference)	0
	Positive	-0.40245066
**HER2 status**	Negative (reference)	0
	Positive	-0.20705725
**Oestrogen receptor status**	Negative (reference)	0
	Positive	-0.22475207
**Cancer stage at diagnosis**	Stage 1 (reference)	0
	Stage 2	0.91589078
	Stage 3	1.7065489
	Stage 4	2.3440632
**Cancer grade**	Well differentiated (reference)	0
	Moderately differentiated	0.29826785
	Poorly or undifferentiated	0.52620672
**HRT use**	No (reference)	0
(within 6 months prior)	Yes	-0.18103964
**Anti-psychotic medication use**		0
(within 6 months prior)		0.21301738
**Constant**		-2.9552694

		Cox proportional hazards model			Competing risks regression model		
Age group	Events / denominator	Harrell's C	Calibration slope	Calibration-in-the-large	Harrell's C*	Calibration slope	Calibration-in-the-large
20-29 years	26 / 317	0.821 (0.719 to 0.906)	1.308 (0.661 to 1.955)	0.308 (-0.339 to 0.955)	0.849 (0.748 to 0.951)	1.553 (0.930 to 2.176)	0.553 (-0.070 to 1.176)
30-39 years	287 / 3,259	0.786 (0.754 to 0.817)	1.196 (1.025 to 1.367)	0.196 (0.025 to 0.367)	0.809 (0.766 to 0.852)	1.317 (1.053 to 1.582)	0.317 (0.053 to 0.582)
40-49 years	865 / 12,398	0.833 (0.818 to 0.848)	1.352 (1.246 to 1.459)	0.352 (0.246 to 0.459)	0.848 (0.828 to 0.867)	1.421 (1.263 to 1.579)	0.421 (0.263 to 0.579)
50-59 years	1,230 / 19,648	0.866 (0.854 to 0.877)	1.264 (1.193 to 1.335)	0.264 (0.193 to 0.335)	0.879 (0.864 to 0.893)	1.287 (1.193 to 1.380)	0.287 (0.193 to 0.380)
60-69 years	1,402 / 20,400	0.859 (0.847 to 0.870)	1.206 (1.140 to 1.273)	0.206 (0.140 to 0.273)	0.872 (0.856 to 0.888)	1.270 (1.166 to 1.374)	0.270 (0.166 to 0.374)
70-79 years	1,840 / 14,443	0.822 (0.811 to 0.833)	1.070 (1.014 to 1.127)	0.070 (0.014 to 0.127)	0.824 (0.807 to 0.841)	1.152 (1.059 to 1.245)	0.152 (0.059 to 0.245)
80+ years	3,158 / 11,915	0.762 (0.752 to 0.772)	0.874 (0.828 to 0.920)	-0.126 (-0.172 to -0.080)	0.740 (0.721 to 0.760)	0.834 (0.758 to 0.909)	-0.166 (-0.242 to -0.091)

Supplementary Table 6. Age group-specific regression model performance metrics (with 95% confidence intervals) estimated after internal-external cross-validation in the data from period 2 (2010-2020). Harrell's C for the competing risks regression model was weighted by the inverse probability of censoring.

		XGBoost			Neural network		
Ethnic group	Events / denominator	Harrell's C*	Calibration slope	Calibration-in-the-large	Harrell's C*	Calibration slope	Calibration-in-the-large
White	5,980 / 58,860	0.819 (0.810 to 0.828)	1.098 (1.058 to 1.139)	0.098 (0.058 to 0.139)	0.797 (0.785 to 0.808)	1.085 (1.053 to 1.118)	0.085 (0.053 to 0.118)
Indian	92 / 1,157	0.839 (0.783 to 0.894)	1.251 (0.943 to 1.560)	0.251 (-0.057 to 0.560)	0.840 (0.770 to 0.910)	1.227 (0.970 to 1.484)	0.227 (-0.030 to 0.484)
Pakistani	57 / 634	0.807 (0.717 to 0.897)	1.227 (0.824 to 1.630)	0.227 (-0.176 to 0.630)	0.732 (0.622 to 0.842)	1.079 (0.781 to 1.377)	0.079 (-0.219 to 0.377)
Bangladeshi	24 / 251	0.722 (0.592 to 0.853)	1.306 (0.791 to 1.820)	0.306 (-0.209 to 0.820)	0.738 (0.597 to 0.879)	1.392 (0.940 to 1.844)	0.392 (-0.060 to 0.844)
Other Asian	43 / 761	0.830 (0.735 to 0.925)	1.507 (1.008 to 2.007)	0.507 (0.008 to 1.007)	0.823 (0.715 to 0.930)	1.429 (1.078 to 1.780)	0.429 (0.078 to 0.780)
Black Caribbean	126 / 909	0.797 (0.727 to 0.868)	1.040 (0.781 to 1.300)	0.040 (-0.219 to 0.300)	0.816 (0.740 to 0.891)	1.053 (0.839 to 1.267)	0.053 (-0.161 to 0.267)
Black African	99 / 854	0.806 (0.745 to 0.866)	1.051 (0.757 to 1.345)	0.051 (-0.243 to 0.345)	0.806 (0.733 to 0.879)	0.989 (0.747 to 1.232)	-0.011 (-0.253 to 0.232)
Chinese	9 / 285	0.912 (0.784 to 1.000)	1.862 (1.017 to 2.708)	0.862 (0.017 to 1.708)	0.931 (0.821 to 1.000)	1.704 (1.067 to 2.341)	0.704 (0.067 to 1.341)
Other ethnic group	88 / 1,374	0.809 (0.753 to 0.865)	1.579 (1.230 to 1.927)	0.579 (0.230 to 0.927)	0.827 (0.754 to 0.900)	1.495 (1.214 to 1.776)	0.495 (0.214 to 0.776)

Supplementary Table 7. Ethnic group-specific regression model performance metrics (with 95% confidence intervals) estimated after internal-external cross-validation in the data from period 2 (2010-2020). Event and denominator counts for each ethnic group are from the 'complete case' data for reference, but performance metrics were calculated using the multiply imputed datasets. * = weighted by the inverse probability of censoring.

		XGBoost			Neural network		
Age group	Events / denominator	Harrell's C	Calibration slope	Calibration-in-the-large	Harrell's C*	Calibration slope	Calibration-in-the-large
20-29 years	26 / 317	0.787 (0.649 to 0.924)	0.914 (0.375 to 1.452)	-0.086 (-0.625 to 0.452)	0.840 (0.723 to 0.958)	0.881 (0.459 to 1.304)	-0.119 (-0.541 to 0.304)
30-39 years	287 / 3,259	0.714 (0.660 to 0.769)	1.316 (1.049 to 1.584)	0.316 (0.049 to 0.584)	0.769 (0.712 to 0.826)	1.017 (0.859 to 1.176)	0.017 (-0.141 to 0.176)
40-49 years	865 / 12,398	0.793 (0.766 to 0.820)	1.276 (1.140 to 1.412)	0.276 (0.140 to 0.412)	0.807 (0.780 to 0.833)	1.243 (1.136 to 1.350)	0.243 (0.136 to 0.350)
50-59 years	1,230 / 19,648	0.866 (0.850 to 0.882)	1.151 (1.074 to 1.228)	0.151 (0.074 to 0.228)	0.839 (0.820 to 0.859)	1.282 (1.209 to 1.356)	0.282 (0.209 to 0.356)
60-69 years	1,402 / 20,400	0.861 (0.845 to 0.876)	1.246 (1.153 to 1.338)	0.246 (0.153 to 0.338)	0.833 (0.814 to 0.851)	1.319 (1.233 to 1.405)	0.319 (0.233 to 0.405)
70-79 years	1,840 / 14,443	0.802 (0.787 to 0.818)	1.099 (1.005 to 1.194)	0.099 (0.005 to 0.194)	0.769 (0.749 to 0.788)	1.053 (0.986 to 1.120)	0.053 (-0.014 to 0.120)
80+ years	3,158 / 11,915	0.708 (0.687 to 0.730)	0.783 (0.719 to 0.847)	-0.217 (-0.281 to -0.153)	0.682 (0.659 to 0.704)	0.641 (0.595 to 0.687)	-0.359 (-0.405 to -0.314)

Supplementary Table 8. Age group-specific regression model performance metrics (with 95% confidence intervals) estimated after internal-external cross-validation in the data from period 2 (2010-2020). Harrell's C for the competing risks regression model was weighted by the inverse probability of censoring.

		Cox proportional hazards model			Competing risks regression		
Stage at diagnosis	Events / denominator	Harrell's C	Calibration slope	Calibration-in-the-large	Harrell's C*	Calibration slope	Calibration-in-the-large
Stage I	786 / 23,423	0.820 (0.804 to 0.834)	1.375 (1.285 to 1.465)	0.375 (0.285 to 0.465)	0.842 (0.818 to 0.866)	1.076 (1.013 to 1.138)	0.076 (0.013 to 0.138)
Stage II	2,067 / 21,473	0.776 (0.767 to 0.786)	1.191 (1.135 to 1.246)	0.191 (0.135 to 0.246)	0.796 (0.780 to 0.812)	1.314 (1.232 to 1.397)	0.314 (0.232 to 0.397)
Stage III	1,156 / 4,948	0.744 (0.731 to 0.757)	0.999 (0.929 to 1.069)	-0.001 (-0.071 to 0.069)	0.761 (0.736 to 0.786)	1.039 (0.833 to 1.244)	0.039 (-0.167 to 0.244)
Stage IV	1,708 / 3,011	0.713 (0.700 to 0.726)	0.803 (0.736 to 0.871)	-0.197 (-0.264 to -0.129)	0.681 (0.658 to 0.704)	0.837 (0.557 to 1.119)	-0.162 (-0.443 to 0.119)
		**XGBoost**			**Neural network**		
Stage I	786 / 23,423	0.810 (0.780 to 0.840)	1.535 (1.404 to 1.666)	0.535 (0.404 to 0.666)	0.756 (0.721 to 0.791)	1.684 (1.567 to 1.800)	0.684 (0.567 to 0.800)
Stage II	2,067 / 21,473	0.766 (0.747 to 0.786)	1.185 (1.121 to 1.249)	0.185 (0.121 to 0.249)	0.757 (0.735 to 0.780)	1.117 (1.066 to 1.168)	0.117 (0.066 to 0.168)
Stage III	1,156 / 4,948	0.678 (0.643 to 0.713)	0.614 (0.503 to 0.725)	-0.386 (-0.497 to -0.275)	0.676 (0.633 to 0.719)	0.602 (0.510 to 0.694)	-0.398 (-0.490 to -0.306)
Stage IV	1,708 / 3,011	0.620 (0.590 to 0.649)	0.276 (0.139 to 0.412)	-0.724 (-0.861 to -0.588)	0.621 (0.593 to 0.650)	0.126 (0.005 to 0.247)	-0.874 (-0.995 to -0.753)

Supplementary Table 9. Tumour stage-specific regression model performance metrics (with 95% confidence intervals) estimated after internal-external cross-validation in the data from period 2 (2010-2020). Event and denominator counts for each ethnic group are from the 'complete case' data for reference, but performance metrics were calculated using the multiply imputed datasets. * = weighted by the inverse probability of censoring.

Cohort group (n)	Crude mortality rate per 10,000 person-years (95% confidence interval)
Overall study cohort (n=141,765)	295.79 (291.75 to 299.88)
Patients with stage missing (n=68,175)	331.21 (325.09 to 337.45)
Patients with progesterone receptor status missing (n=112,258)	294.29 (289.85 to 298.80)
Patients with oestrogen receptor status missing (n=89,139)	302.29 (297.28 to 307.40)
Patients with HER2 receptor status missing (n=92,955)	304.53 (299.63 to 309.50)

Supplementary Table 10. Crude breast cancer-related mortality rates in different sub-sets of the cohort, based on data missingness. Follow-up was calculated from date of breast cancer diagnosis to the earliest of: date of breast cancer death, date of death from another cause, censoring date (entry + 10 years), or leaving the general practice.
