# Supplementary material for: Development and internal-external validation of statistical and machine learning models for breast cancer prognostication: cohort study
Source: BMJ. 2023 May 10;381:e073800. doi: 10.1136/bmj-2022-073800 (PMC10170264; doi:10.1136/bmj-2022-073800)

Supplementary Figure 1 – Unadjusted mortality curves by ethnic group in the study cohort.

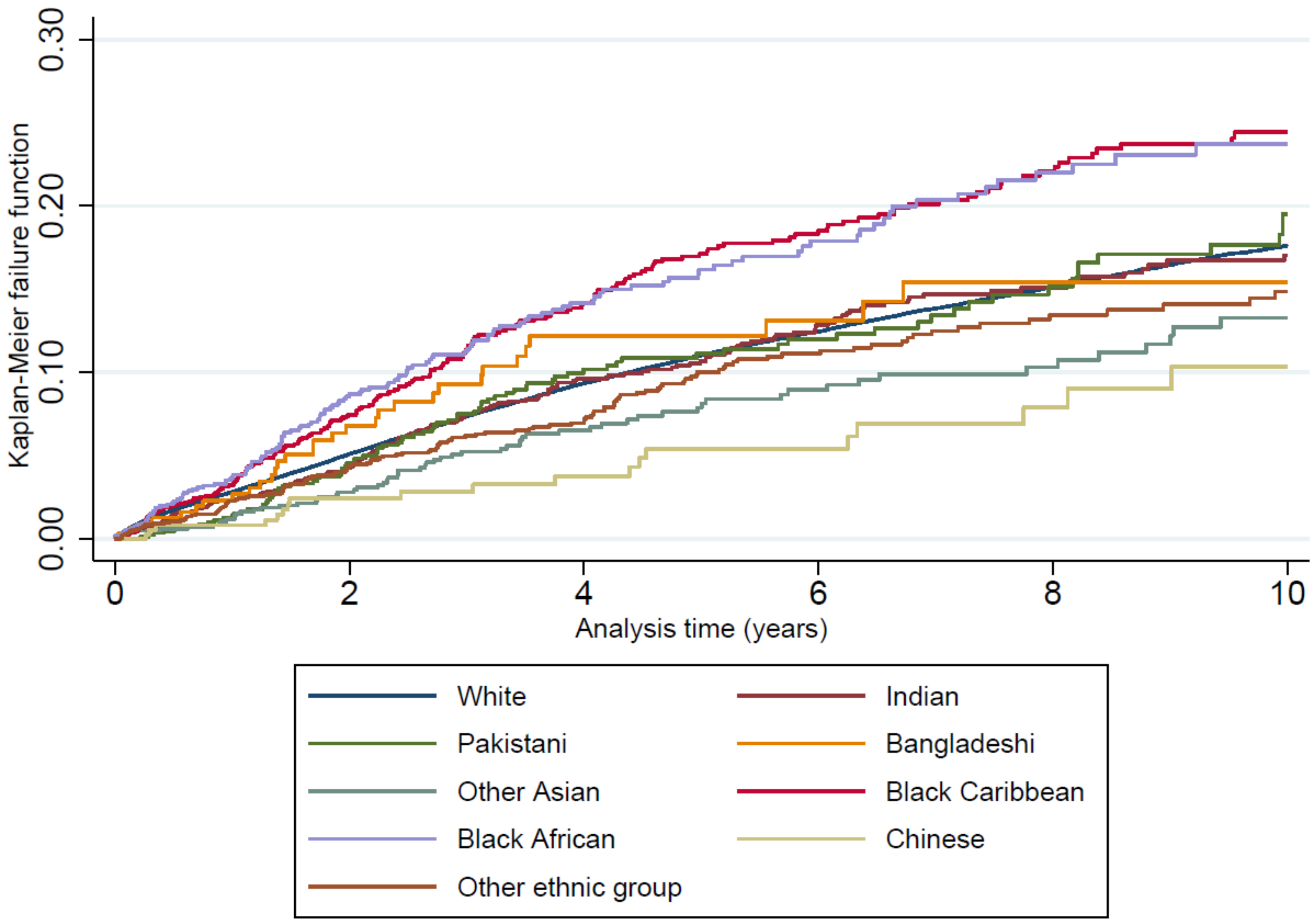

Supplementary Figure 2 – Fractional polynomial terms for age and body mass index, for the Cox model (top row), and Competing Risks Regression models (bottom row).

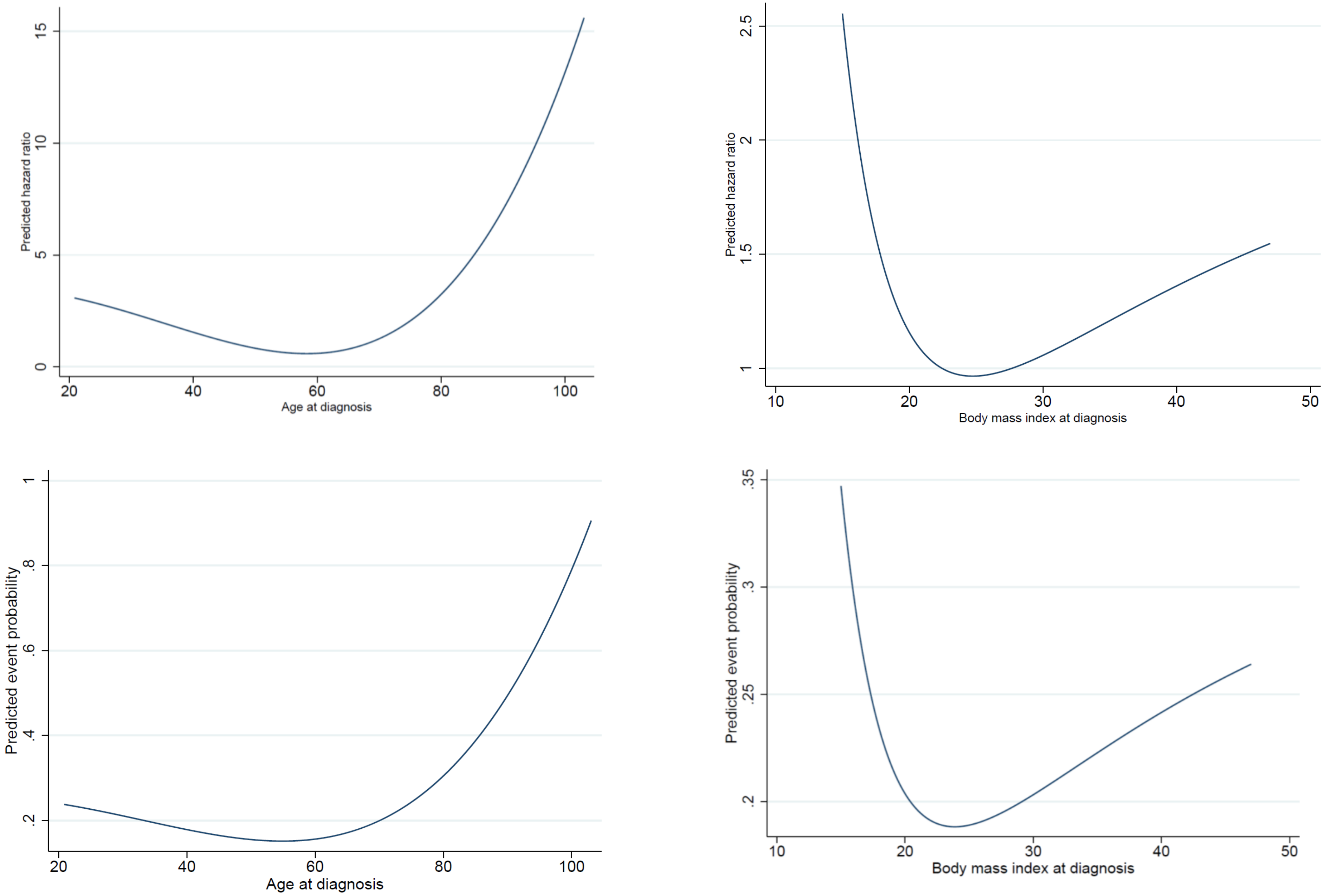

Supplementary Figure 3 – Performances metrics for the Competing risks regression model, estimated after internal-external cross-validation.

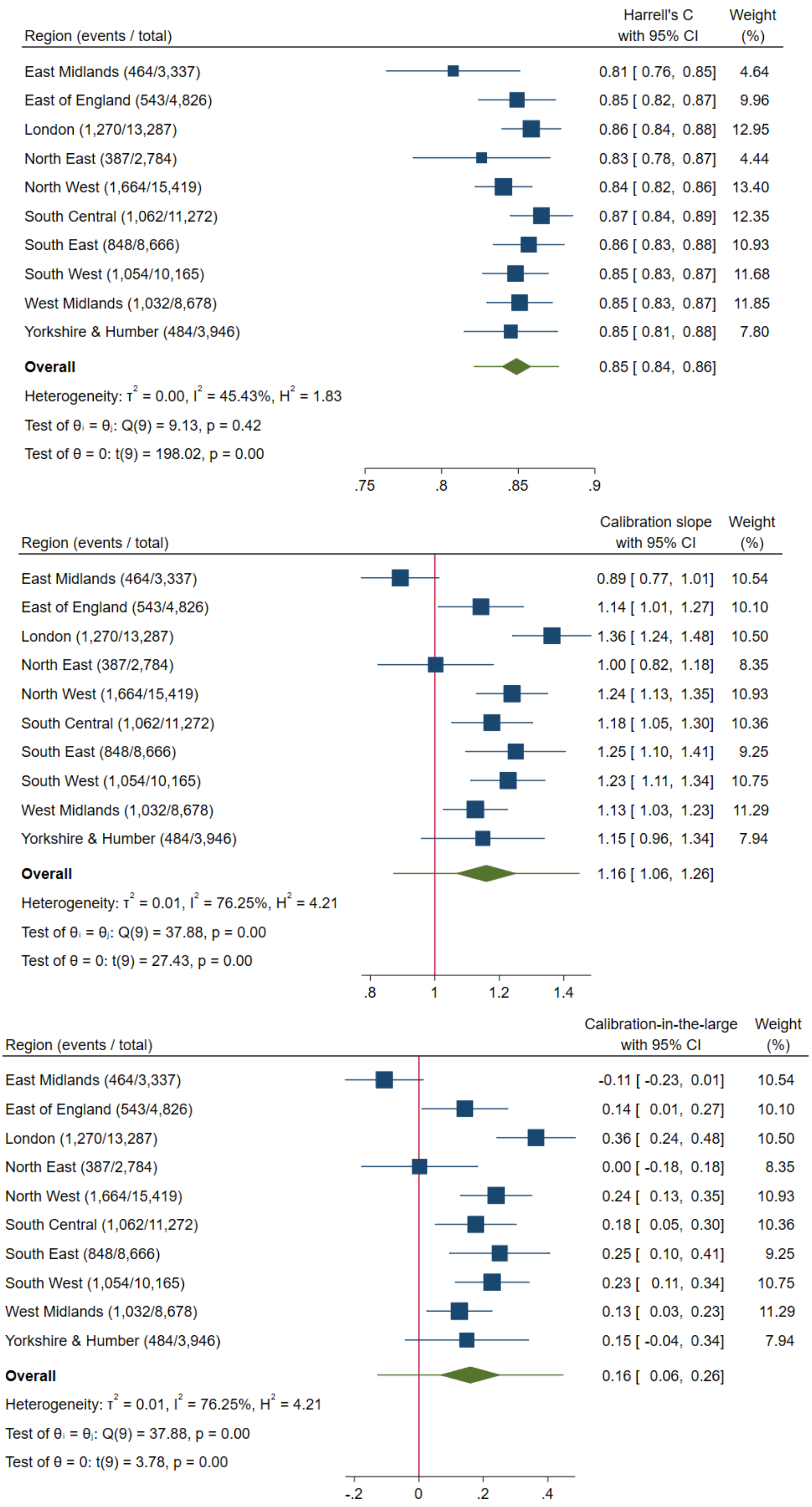

Supplementary Figure 4 – Performance metrics for the XGBoost model estimated after internal-external cross-validation.

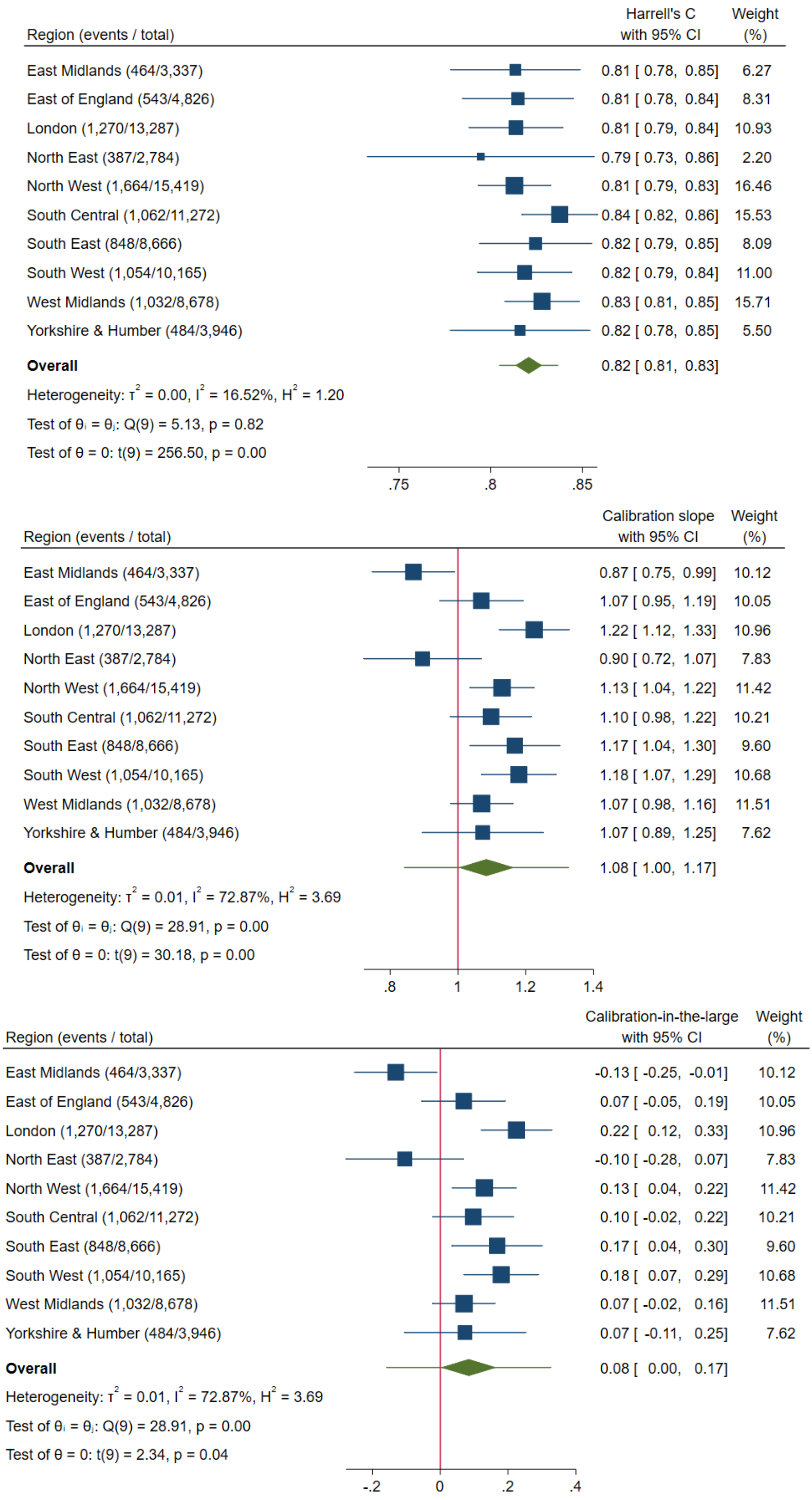

Supplementary Figure 5 – Performance metrics for the neural network estimated after internal-external cross-validation**.**

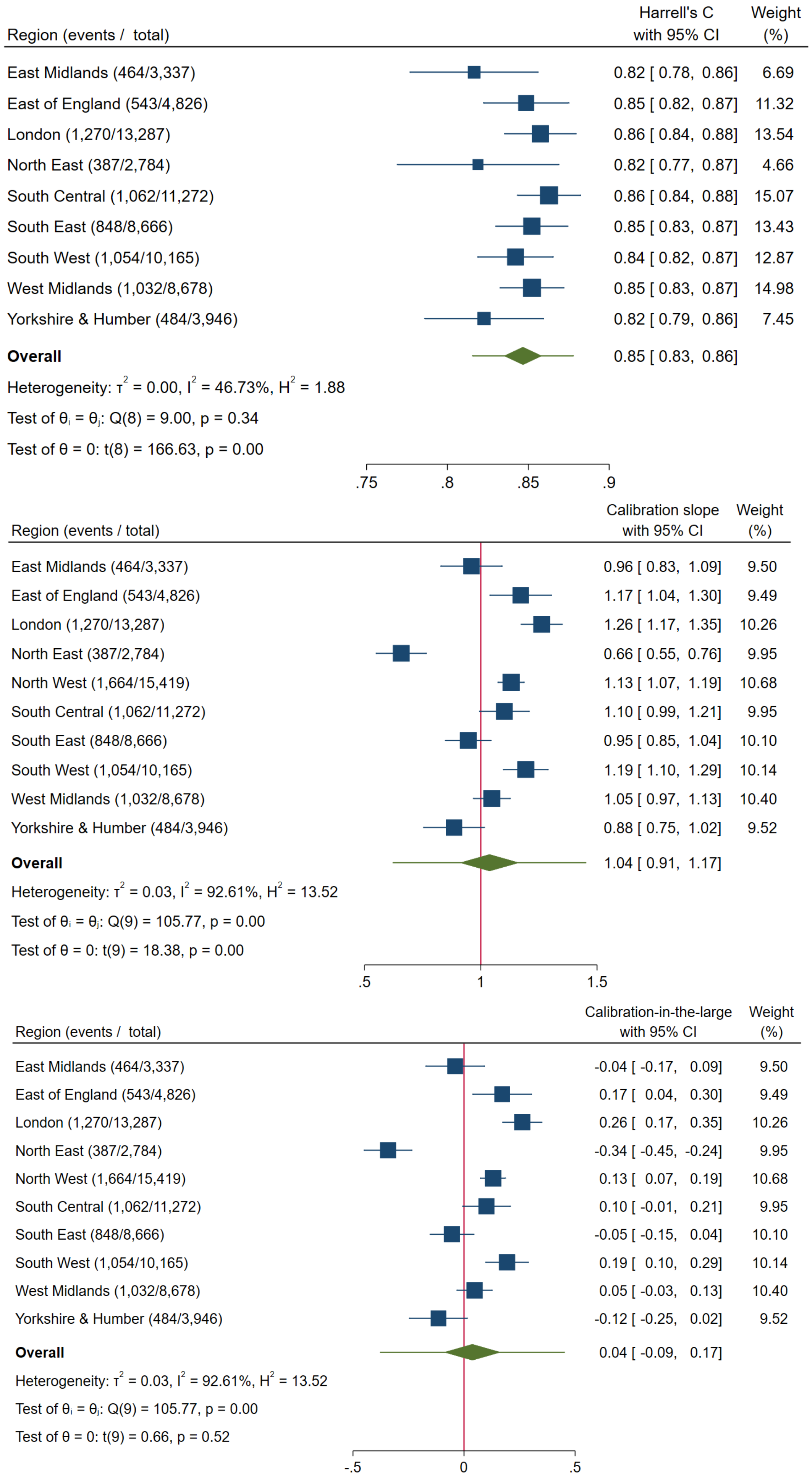

Supplementary Figure 6. Decision curve analysis displaying clinical utility of all 4 models, by cancer stage at diagnosis.

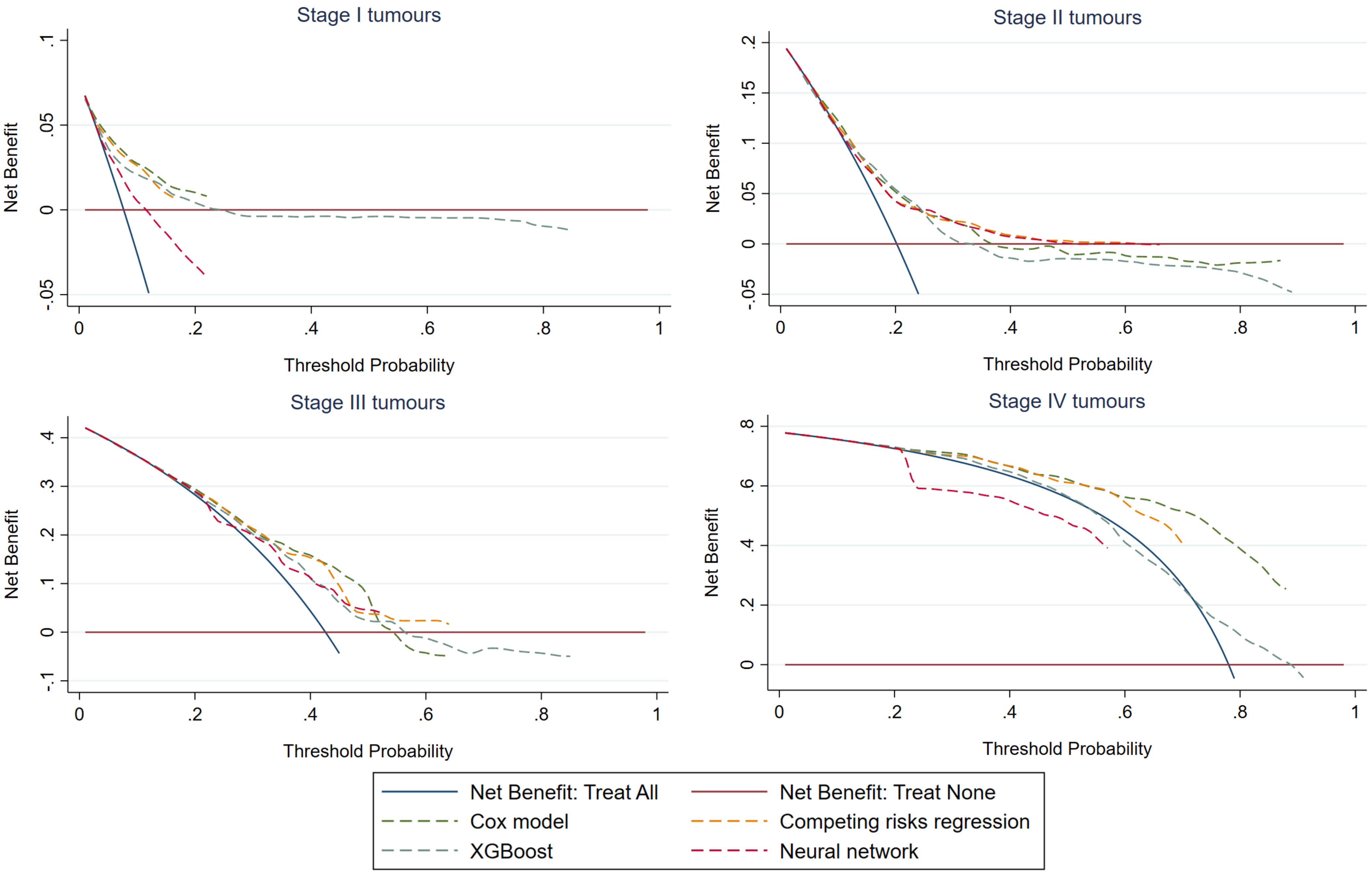

Supplementary Figure 7. Comparison of predictions from the final Cox proportional hazards model and the three competing risks models (regression [top], XGBoost [middle] and neural network [bottom].

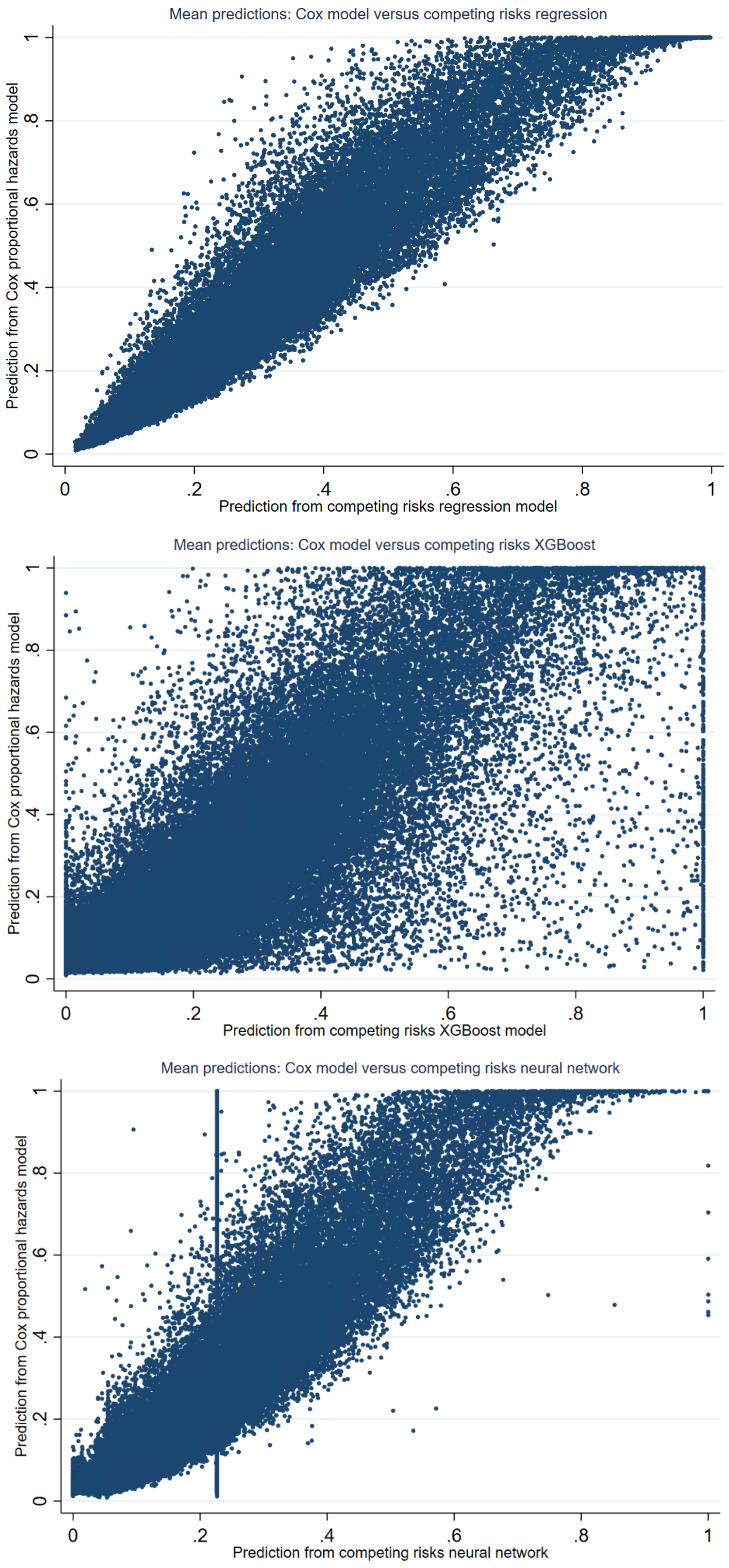

Supplementary Figure 8. Comparison of predictions from the final competing risks regression model and the competing risks XGBoost (top) and neural network [bottom] models.

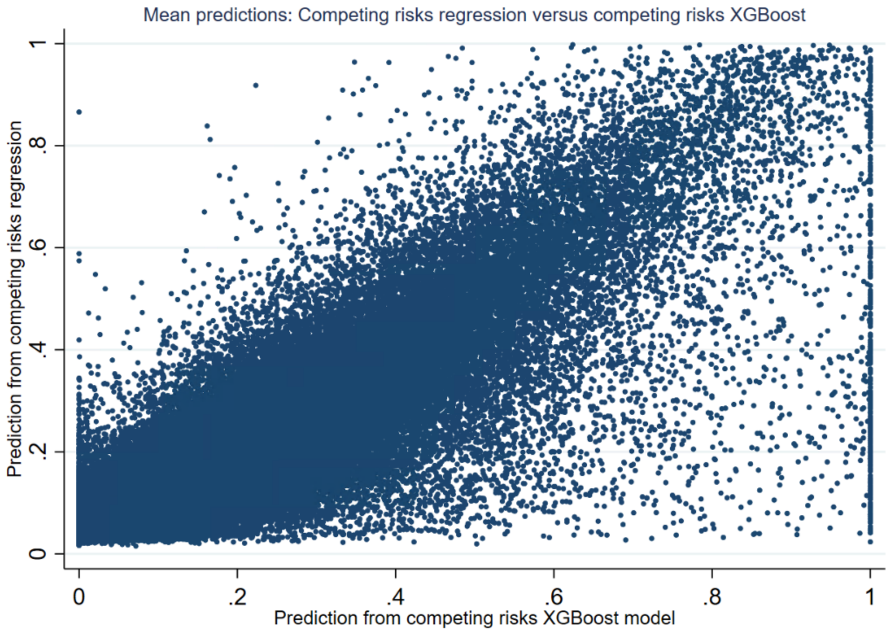

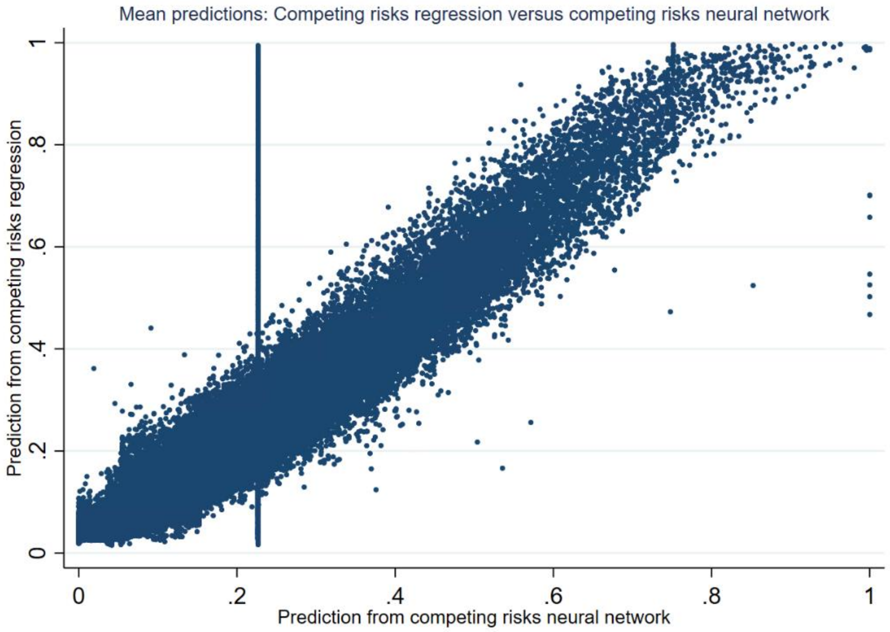

Supplementary Figure 9. Relative predictor importance for the final XGBoost model – relative importance was calculated using the 'gain' metric, as produced by the XGBoost package in R.

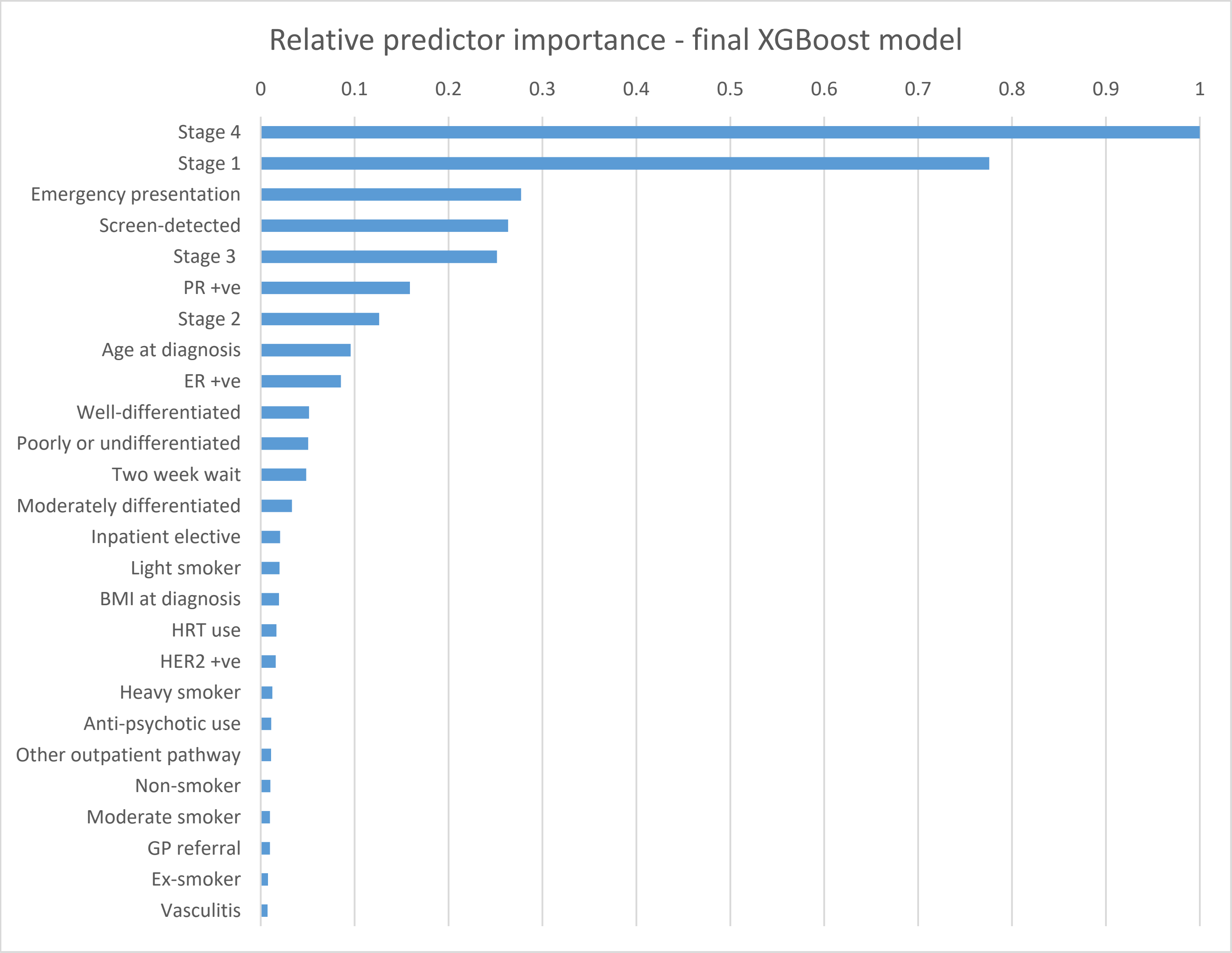

Supplement: Supplementary file 2 — Supplementary information: additional figures 1-9 [file clia073800.ww2.pdf]
